# Comparative transcriptomic analysis of *Rickettsia conorii* during in vitro infection of human and tick host cells

**DOI:** 10.1186/s12864-020-07077-w

**Published:** 2020-09-25

**Authors:** Hema P. Narra, Abha Sahni, Jessica Alsing, Casey L. C. Schroeder, George Golovko, Anna M. Nia, Yuriy Fofanov, Kamil Khanipov, Sanjeev K. Sahni

**Affiliations:** 1 Department of Pathology, University of Texas Medical Branch, Galveston, TX 77555 USA; 2 Department of Pharmacology and Toxicology, University of Texas Medical Branch, Galveston, TX 77555 USA; 3 Biochemistry and Molecular Biology, University of Texas Medical Branch, Galveston, TX 77555 USA

**Keywords:** *Rickettsia*, Endothelial cells, Tick vector, Small RNAs, 6S RNA, Transcriptome, Transcription start sites, and Terminator 5′-phosphate-dependent exonuclease (TEX)

## Abstract

**Background:**

Pathogenic *Rickettsia* species belonging to the spotted fever group are arthropod-borne, obligate intracellular bacteria which exhibit preferential tropism for host microvascular endothelium in the mammalian hosts, resulting in disease manifestations attributed primarily to endothelial damage or dysfunction. Although rickettsiae are known to undergo evolution through genomic reduction, the mechanisms by which these pathogens regulate their transcriptome to ensure survival in tick vectors and maintenance by transovarial/transstadial transmission, in contrast to their ability to cause debilitating infections in human hosts remain unknown. In this study, we compare the expression profiles of rickettsial sRNAome/transcriptome and determine the transcriptional start sites (TSSs) of *R. conorii* transcripts during in vitro infection of human and tick host cells.

**Results:**

We performed deep sequencing on total RNA from *Amblyomma americanum* AAE2 cells and human microvascular endothelial cells (HMECs) infected with *R. conorii*. Strand-specific RNA sequencing of *R. conorii* transcripts revealed the expression 32 small RNAs (*Rc*_sR’s), which were preferentially expressed above the limit of detection during tick cell infection, and confirmed the expression of *Rc*_sR61, sR71, and sR74 by quantitative RT-PCR. Intriguingly, a total of 305 and 132 *R. conorii* coding genes were differentially upregulated (> 2-fold) in AAE2 cells and HMECs, respectively. Further, enrichment for primary transcripts by treatment with Terminator 5′-Phosphate-dependent Exonuclease resulted in the identification of 3903 and 2555 transcription start sites (TSSs), including 214 and 181 primary TSSs in *R. conorii* during the infection to tick and human host cells, respectively. Seventy-five coding genes exhibited different TSSs depending on the host environment. Finally, we also observed differential expression of 6S RNA during host-pathogen and vector-pathogen interactions in vitro, implicating an important role for this noncoding RNA in the regulation of rickettsial transcriptome depending on the supportive host niche.

**Conclusions:**

In sum, the findings of this study authenticate the presence of novel *Rc*_sR’s in *R. conorii,* reveal the first evidence for differential expression of coding transcripts and utilization of alternate transcriptional start sites depending on the host niche, and implicate a role for 6S RNA in the regulation of coding transcriptome during tripartite host-pathogen-vector interactions.

## Background

Human pathogens in the family *Rickettsiaceae* include Gram-negative bacteria capable of establishing an intracellular habitat as obligate intracellular parasites to derive energy and nutrients from the host cytosol for their growth, replication, and dissemination. Rickettsial infections associated with significant morbidity and mortality constitute a significant health scourge across the globe [1, 2]. Mediterranean spotted fever due to *Rickettsia conorii* is an acute febrile zoonotic disease with flu-like initial symptoms and typically associated with eschars at the bite sites of tick vectors [3]. Transovarial and transstadial transmission are considered to be the major driving forces for *R. conorii* maintenance and persistence in its natural arthropod vectors [3]. In its mammalian hosts, including humans, *R. conorii* exhibits tropism for human microvascular endothelium lining the small or medium-sized blood vessels leading to vascular inflammation and dysfunction manifesting as increased vascular permeability, fluid imbalance, and edema of vital organ systems [4].

Transcriptional and epigenetic regulation of the transcriptome is presumed to play a vital role in rickettsial homeostasis during their transition and establishment in homoeothermic mammalian hosts vis-à-vis tick (poikilothermic) vectors. Limited transcriptional changes occurring due to a shift in growth temperature (37 °C vs 25 °C), iron limitation, or infection of different host cell species in vitro have been reported for *R. rickettsii*. Microarray based transcriptomic analysis of the typhus group pathogen *R. typhi* grown at different temperatures has shown up- and down-regulation of a total of 70 and 60 genes upon temperature shift from 37 °C to 25 °C, respectively [5, 6]. Interestingly, 56 genes are differentially regulated in *R. rickettsii* in response to a cold shock (4 °C), indicating the intrinsic ability of this pathogen to respond to changes in environmental cues, in vitro [5]. On the other hand, about 13% of *R. rickettsii* genes are differentially modulated by temperature upshift from 25 °C to 35 °C, and acquisition of blood meal by tick vectors. Notably, while genes involved in DNA replication, recombination and repair, vesicular transport and secretion, and energy production and conversion display induced expression, a majority of genes involved in translation, ribosomal structure, and biogenesis pathways are downregulated during the process of tick feeding [7]. In addition, rickettsial gene expression is also influenced by tick gender and organ of colonization. Nearly 67 and 80% of the 85 rickettsial genes tested have been reported to be differentially expressed in salivary glands and midguts, respectively. While genes encoding type IV secretion system were exclusively induced in females during rickettsial infection, co-chaperone HscB, and thioredoxin peroxidase 1 were expressed only in male ticks [8]. Together, these findings signify the importance of blood feeding, colonizing organ, and tick gender on rickettsial gene expression as the potential basis for altered virulence during natural transmission from the transmitting vector to the mammalian hosts.

The effect of host environment (human vis-à-vis arthropod) on bacterial transcriptional landscape has been documented for several other vector-borne pathogens, including *Borrelia*, *Anaplasma*, and *Ehrlichia* [9–11]. Comparative transcriptomic analysis of *A. phagocytophilum* grown in human (HL-60) and tick (ISE6) cells results in differential expression of 41.5% of the genes, of which 117 exhibit greater than two-fold change [12]. In *Borrelia*, OspA is highly expressed during its colonization in ticks, and OspC is upregulated during tick feeding and transmission, leading to the hypothesis that warm host blood and changes in the temperature during feeding act as a trigger for the modulation of gene expression [13, 14]. Further, *Borrelia* OspB mutants exhibit impaired ability to adhere to gut tissues and survive in tick vectors despite their ability to infect and persist in mice [15]. In contrast, other borrelial genes, namely OspE/F, Arp, P47, and P66, are highly expressed in an infected mammalian host and implicated in host defense and colonization of the vertebrate host [16, 17]. Collectively, these studies suggest an important role for the host environment on the changes in the regulation of transcriptional expression in bacterial pathogens.

Riboregulation of bacterial coding transcriptome by small non-coding RNAs (sRNAs) is being increasingly recognized within the past few years. In this context, several bacterial sRNAs have now been identified to be differentially expressed depending on the host niche, stress conditions, as well as specific growth requirements, and important roles for these sRNAs in cellular networks and transcriptional regulatory circuits have been documented [18, 19]. Nearly 45% of *Pseudomonas putida* sRNAs have been projected to be differentially regulated during osmotic and oxidative stress conditions [20]. Interestingly, PinT, a PhoP activated sRNA of *Salmonella*, not only regulates bacterial coding transcriptome required for the invasion and internalization during in vivo infection, but is also involved in the regulation of several other genes essential for the activation of host cell JAK-STAT signaling pathway and expression of long non-coding RNAs, thus exemplifying important contributory roles for a bacterial small RNA in the regulation of eukaryotic host responses [21]. In recent years, we have applied a combinatorial strategy involving computational and deep sequencing approaches to identify, validate, and characterize bona fide sRNAs and their target genes in *Rickettsia* species belonging to both spotted fever and typhus groups [22–24]. Additionally, RNA sequencing of *R. prowazekii* transcriptome during in vitro infection of human versus tick cells as the host revealed differential expression of coding transcripts and sRNAs in a host-niche specific manner [25].

In the present study, we report the comparative analysis of *R. conorii* transcriptome during the infection of cultured human endothelial cells and tick cells as the host. Deep sequencing of bacterial coding and non-coding transcripts revealed differential expression of several genes dependent on the supportive host cell. Approximately 19% of *R. conorii* genes were differentially and highly expressed during tick cell infection, whereas only 8% of the genes were highly expressed in host HMECs. Overall, a greater number of genes were expressed above the limit of detection during tick cell infection when compared to HMECs. We have also identified 32 *Rc*_sRs to be abundantly expressed during tick AAE2 cell infection when compared to HMECs and validated their expression during in vitro infection of both HMECs and tick cells. We have further determined the differential expression of *R. conorii* 6S RNA depending on the host niche, allowing us to implicate a role for this bacterial sRNA in the regulation of coding transcriptome during host-pathogen and vector-pathogen interactions.

## Results

### Coding transcriptome of *R. conorii* during in vitro infection of human ECs versus tick cells

To decode the transcriptional landscape of *R. conorii* during in vitro infection of human and tick cells as the host, we performed deep sequencing on enriched bacterial RNA isolated from HMECs and AAE2 cells infected with *R. conorii* for 24 h. The rationale for selecting this time point was to allow for adhesion and internalization known to occur almost instantaneously followed by two cycles of replication by intracellular rickettsiae based on their replication time of 9 to 11 h [4]. To ensure valid comparison between host cells under study, we determined the levels of internalized *R. conorii* in human and tick cells and observed similar levels of infection (Additional file 1). We sequenced an average of about 73 million reads from both HMECs as well as AAE2 cells infected with *R. conorii*, of which 26.7 and 18.5% mapped to *R. conorii* genome, respectively. From a total of 1579 annotated coding genes, only 21 genes, including 19 designated as encoding for hypothetical proteins and annotated only by PATRIC, were expressed below the limit of detection in both cell types. The remaining two genes annotated as *RC0419* and *RC0453* are present in *R. conorii* in the PATRIC as well as NCBI databases for sequenced rickettsial genomes (Additional file 2). In addition, 7 relatively small genes ranging between 102 and 144 bp and annotated only by PATRIC were expressed in HMECs, but not in AAE2 cells. Conspicuously, a much larger repertoire of 125 genes, of which 64 have been annotated by both PATRIC and NCBI, were expressed only in tick cells. These included genes coding for integration host factor beta subunit (*RC0757*), competence protein F homolog (*peg.961*), Bcr/CflA family multidrug resistance transporter (*peg.1053*), toxin-antitoxin system (*RC0914* and *RC1143*), and plasmid maintenance system antidote protein (*peg.521*), in addition to a considerable number (61) of transcripts coding for hypothetical proteins with unknown function (Additional file 2).

Interestingly, N-acetylmuramoyl-L-alanine amidase (*RC0497*) was determined to be the most abundantly expressed transcript during the infection of both tick and human host cells (Tables 1 and 2). The outer membrane protein B (*rompB*), heat shock protein 60 family chaperones (*groEL* and *groES*), cold shock protein (*cspA*), antitoxins of *relE* (*RC1223*) and *vapC* (*vapB*), tol-pal system peptidoglycan-associated protein (*pal*), CarD like transcription factor (*carD*), translation elongation factor Tu (*tuf*), and *rickA* involved in actin based motility were among the top 20 *R. conorii* genes highly expressed during infection of tick and host cells (Tables 1 and 2). To further identify *R. conorii* genes differentially expressed during human endothelium versus tick cell infection, we conducted a comparative analysis of the normalized gene expression datasets. The findings revealed that 305 genes were highly expressed (log_2_ fold change ≥2.0) in tick AAE2 cells, whereas only 132 genes displayed differential upregulation in human ECs (Additional file 2). A majority (~ 90%) of top 20 differentially and highly expressed genes in tick AAE2 cells and HMECs encoded hypothetical proteins with putative functions (Tables 3 and 4). BLASTp analysis revealed that while genes predicted to encode for tetratricopeptide repeat proteins, acid phosphatase, metalloprotease, and transcriptional regulator activities were distinctly upregulated in tick cells, those likely involved in transport, toxin-antitoxin system, nucleotide synthesis, and membrane proteins were abundantly expressed in HMECs (Tables 3 and 4). *R. conorii* RC0446 and RC0511, presumed to function respectively as an M61 glycyl aminopeptidase and AbrB family transcriptional regulator, and peg.0696 with a hypothetical function, were the top three differentially and highly expressed genes in tick cells (log_2_ fold change > 5.0). On the other hand, RC0257, coding for a putative autotransporter outer membrane beta-barrel domain containing protein was the only gene upregulated at a threshold of > 5.0 log_2_ fold during *R. conorii* infection of HMECs (Tables 3 and 4; Additional file 2).

**Table 1:** List of top 20 *R. conorii* genes highly expressed during the infection of tick vector cells, in vitro

Gene ID	Gene Name	Product	Gene Length	TPM
RC0497		N-acetylmuramoyl-L-alanine amidase	804	43,824
RC0909	*rickA*	Involved in actin based motility	1554	12,329
RC1223		Antitoxin to RelE-like translational repressor toxin	429	8472
RC0813		Hypothetical protein	237	8391
RC1085	*rompB*	Outer membrane protein B	4968	8203
RC1008	*tuf*	Translation elongation factor Tu	1185	7505
RC0968	*groEL*	Heat shock protein 60 family chaperone GroEL	1647	7137
RC1021	*cspA*	Cold shock protein of CSP family	213	6689
RC0207		Conserved hypothetical protein	954	6560
RC1200	*pal*	Tol-Pal system peptidoglycan-associated lipoprotein	468	6137
RC0876		Uncharacterized protein RT0563	210	6002
RC0969	*groES*	Heat shock protein 60 family co-chaperone GroES	288	5353
RC0858		Cell division protein MraZ	450	5294
RC1071		type II toxin-antitoxin system HicB family antitoxin	345	5105
RC0030	*carD*	CarD-like transcriptional regulator	522	5033
RC0380	*vapB*	VapB protein (antitoxin to VapC)	288	4949
RC1020	*rhlE*	DEAD-box ATP-dependent RNA helicase CshA	1245	4601
RC0060		Hypothetical protein	492	4285
peg.1340		Hypothetical protein	129	4197
RC0649		Hypothetical protein	183	4054

*TPM* Transcripts per million

**Table 2:** List of top 20 *R. conorii* genes highly expressed during the infection of human host cells, in vitro

Gene ID	Gene Name	Product	Gene Length	TPM
RC0497		N-acetylmuramoyl-L-alanine amidase	804	28,819
RC0949	*rpsU*	SSU ribosomal protein S21p	201	21,374
RC1021	*cspA*	Cold shock protein of CSP family	213	15,638
RC0909	*rickA*	Involved in actin based motility	1554	14,586
RC0813		Hypothetical protein	237	14,037
RC0277		Hypothetical protein	216	11,593
RC1223		Antitoxin to RelE-like translational repressor toxin	429	9342
RC0207		Hypothetical protein	954	8478
RC0968	*groEL*	Heat shock protein 60 family chaperone GroEL	1647	8364
RC1008	*tuf*	Translation elongation factor Tu	1185	8315
RC1085	*rompB*	Outer membrane protein B	4968	8300
RC1200	*pal*	Tol-Pal system peptidoglycan-associated lipoprotein	468	7930
RC0876		Uncharacterized protein RT0563	210	7902
RC0152	*rplS*	LSU ribosomal protein L19p	417	7473
RC1362		Hypothetical protein	165	6888
RC0060		Hypothetical protein	492	6643
RC0315	*rplM*	LSU ribosomal protein L13p (L13Ae)	468	6487
RC0668		Hypothetical protein	378	6398
RC0030		CarD-like transcriptional regulator	522	6376
RC0380	*vapB*	VapB protein (antitoxin to VapC)	288	6320

*TPM* Transcripts per million

**Table 3:** List of top 20 *R. conorii* genes differentially expressed during the infection of tick vector AAE2 cells, in vitro

Gene ID	Product	Gene Length (bp)	TPM		Log_**2**_ Change	Homologous to			
			AAE2 + ***Rc***	HMEC + ***Rc***		Product	Organism	E-value	Identity (%)
RC0446	Hypothetical protein	291	377	8	5.64	M61 glycyl aminopeptidase family protein	*R. rhipicephali*	6e-52	92%
RC0511	Hypothetical protein	249	367	9	5.38	Transcriptional regulator, AbrB family	*R. massiliae*	1e-50	100%
peg.0696	Hypothetical protein	132	539	17	5.02	–	–	–	–
peg.1306	Hypothetical protein	114	415	19	4.43	Acetyl-CoA acetyltransferase	Thermoplasmata	1e-05	48%
RC0418	Hypothetical protein	672	119	7	4.18	Tetratricopeptide repeat family protein	*R. hoogstraalii*	2e-121	81%
RC1248	Hypothetical protein	153	234	14	4.03	HEPN domain-containing protein	*R. asembonensis*	2e-07	92%
peg.0362	Hypothetical protein	117	297	19	3.98	–	–	–	–
peg.0481	Hypothetical protein	198	338	22	3.93	CPBP family intramembrane metalloprotease	*R. gravesii*	4e-35	93%
RC0253	Hypothetical protein	270	343	24	3.81	–	–	–	–
RC0979	DnaA regulatory inactivator Hda (Homologous to DnaA)	669	138	10	3.81				
RC0034	Dihydrofolate reductase (FolA)	495	62	4	3.8				
RC0570	RND efflux system, membrane fusion protein	297	282	22	3.67				
RC1250	Hypothetical protein	246	417	36	3.54	Palindromic element RPE1 domain-containing protein	SFG rickettsiae	7e-51	100%
RC0640	Hypothetical protein	420	304	26	3.54	Fimbrial biogenesis outer membrane usher protein	*R. gravesii*	2e-65	96%
RC0957	Hypothetical protein	201	245	22	3.49	Tetratricopeptide repeat protein	SFG rickettsiae	1e-17	97%
RC0471	Hypothetical protein	249	193	18	3.45	Tetratricopeptide repeat protein	*R. hoogstraalii*	7e-46	95%
RC0513	Hypothetical protein	186	388	35	3.45	–	–	–	–
peg.0400	Hypothetical protein	144	332	31	3.44	Acid phosphatase family protein	*R. amblyommatis*	5e-26	94%
RC1293	Hypothetical protein	195	363	34	3.43	–	–	–	–
peg.0013	Hypothetical protein	135	172	16	3.4	Toxin	*R. tamurae*	0.086	90%

Only *R. conorii* proteins annotated as ‘hypothetical protein’ were searched by BLASTp to identify the homologous protein in other bacteria

“-“indicates no known homolog was identified in a BLASTp search

*TPM* Transcripts per million

Log2 Change = Log_2_(TPM of AAE2 + *Rc* / TPM of HMEC+*Rc*)

**Table 4:** List of top 20 *R. conorii* genes differentially expressed during the infection of human microvascular endothelial cells, in vitro

Gene ID	Product	Gene Length (bp)	TPM		Log2 Change	Homologous to			
			AAE2 + Rc	HMEC + Rc		Product	Organism	E-value	Identity (%)
RC0257	Hypothetical protein	135	8	743	6.52	Autotransporter outer membrane beta-barrel domain-containing protein	*Citrobacter farmeri*	7e-04	15%
peg.1116	Hypothetical protein	114	10	263	4.78	Sugar (and other) transporter family protein	*R. hoogstraalii*	2e-14	92
peg.0736	Hypothetical protein	186	6	130	4.47	Spore Coat Protein U domain protein	*R. hoogstraalii*	2e-21	89
peg.0341	Hypothetical protein	129	25	522	4.36	ATP-binding cassette domain-containing protein	*R. fournieri*	3e-13	92
RC0682	LSU ribosomal protein L36p, zinc-independent (RpmJ)	126	26	494	4.25				
RC0395	Hypothetical protein	297	11	187	4.09	–	–	–	–
RC1180	Hypothetical protein	138	24	302	3.67	MFS transporter	Chrysiogenales bacterium	7e-06	39
RC1181	Hypothetical protein	267	45	526	3.55	Putative MFS transporter	*Aeromonas salmonicida*	6e-22	34
peg.0129	Hypothetical protein	120	27	311	3.51	ATP synthase A chain	*R. slovaca*	0.009	100
peg.1274	Hypothetical protein	114	57	546	3.25	–	–	–	–
RC0196	Hypothetical protein	174	13	114	3.18	Nucleoside triphosphate pyrophosphohydrolase	*M. smegmatis*	5e-17	45
RC1177	Hypothetical protein	240	32	265	3.06	MFS transporter	*R. felis*	5e-30	84
RC1123	Hypothetical protein	156	7	56	3.01	Myotubularin-related protein 10-B	*Cephus cinctus*	4e-13	27
peg.0810	Hypothetical protein	114	29	218	2.93	Type II toxin-antitoxin system RelB/DinJ family antitoxin	*Psychrobacter*	4e-10	30
peg.1527	Hypothetical protein	129	42	307	2.86	–	–	–	–
RC0824	Hypothetical protein	282	71	503	2.83	BrnT family toxin	*Limnohabitans*	2e-19	52
RC0149	Acetate kinase (EC 2.7.2.1)	252	9	61	2.82				
RC0530	Rickettsial conserved	207	68	477	2.8	Holliday junction ATP-dependent DNA helicase RuvA	*R. fournieri*	1e-13	97
RC0364	Hypothetical protein	186	176	1109	2.66	Palindromic element RPE1 domain-containing protein	*R. hoogstraalii*	1e-20	80
RC0033	PqqC-like protein	303	75	471	2.64	Pyrroloquinoline quinone biosynthesis protein PqqC	*R. japonica*	6e-34	97

Only *R. conorii* proteins annotated as ‘hypothetical protein’ were searched by BLASTp to identify the homologous protein in other bacteria

“-“indicates no known homolog was identified in a BLASTp search

*TPM*: Transcripts per million

Log2 Change = Log_2_(TPM of HMEC+*Rc* / TPM of AAE2 + *Rc*)

As a follow up to the global profiling above, we next focused on the expression of genes involved in lipopolysaccharide biosynthesis, type IV secretion system and secreted effectors, as well as those coding for the proteins containing ankyrin repeats. Of the 19 analyzed genes involved in LPS biosynthesis and transport, only *RC0486* and *RC1055* encoding for Lipid A core-O-antigen ligase and phosphorylcholine transferase were slightly upregulated (2.21 and 2.34 fold, respectively) during tick cell infection as compared to HMECs. The remaining 17 genes exhibited similar levels (fold changes of < ±2) of transcript abundance in both cell types at 24 h post-infection (Additional file 3A). With the notable exception of *virB7* (*RC0386*) expressed at detectable levels only during the infection of tick cells, all other component genes of type IV secretion system were expressed in both tick and human cells. *RC0144* (*virB6*), *RC0385* (*virB8*), and *RC0388* (*virB9*) were slightly upregulated at 2.5-, 2.9- and 2.6-fold, respectively, during tick cell infection when compared to HMECs (Additional file 3B). Further, similar levels of transcript abundance were evident for all rickettsial effectors barring VapC, known to encode an antitoxin to VapB toxin. VapC was highly upregulated in HMECs as the host (TPM = 5969), as compared to tick cells (TPM = 2855). Amongst all known rickettsial effectors, *rickA* had the highest expression in both cell types. Intriguingly, with the exception of *RC0700* which was slightly upregulated (> 2-fold) in infected HMECs, all other proteins containing ankyrin repeats showed higher transcript abundance in tick AAE2 cells (Additional file 3C). *RC0502* and *RC0877* were highly expressed in tick cells as indicated by an increase of 9.9- and 7.7-fold when compared to HMECs (Additional file 3C). Collectively, comparative transcriptional profile of *R. conorii* during the infection of human ECs and tick cells not only confirmed the expression of multiple genes encoding hypothetical functions, but also revealed significantly higher expression levels of several genes in tick cells as opposed to human ECs.

### Quantitative RT-PCR based validation of differential regulation of coding transcripts

**Fig. 1:**
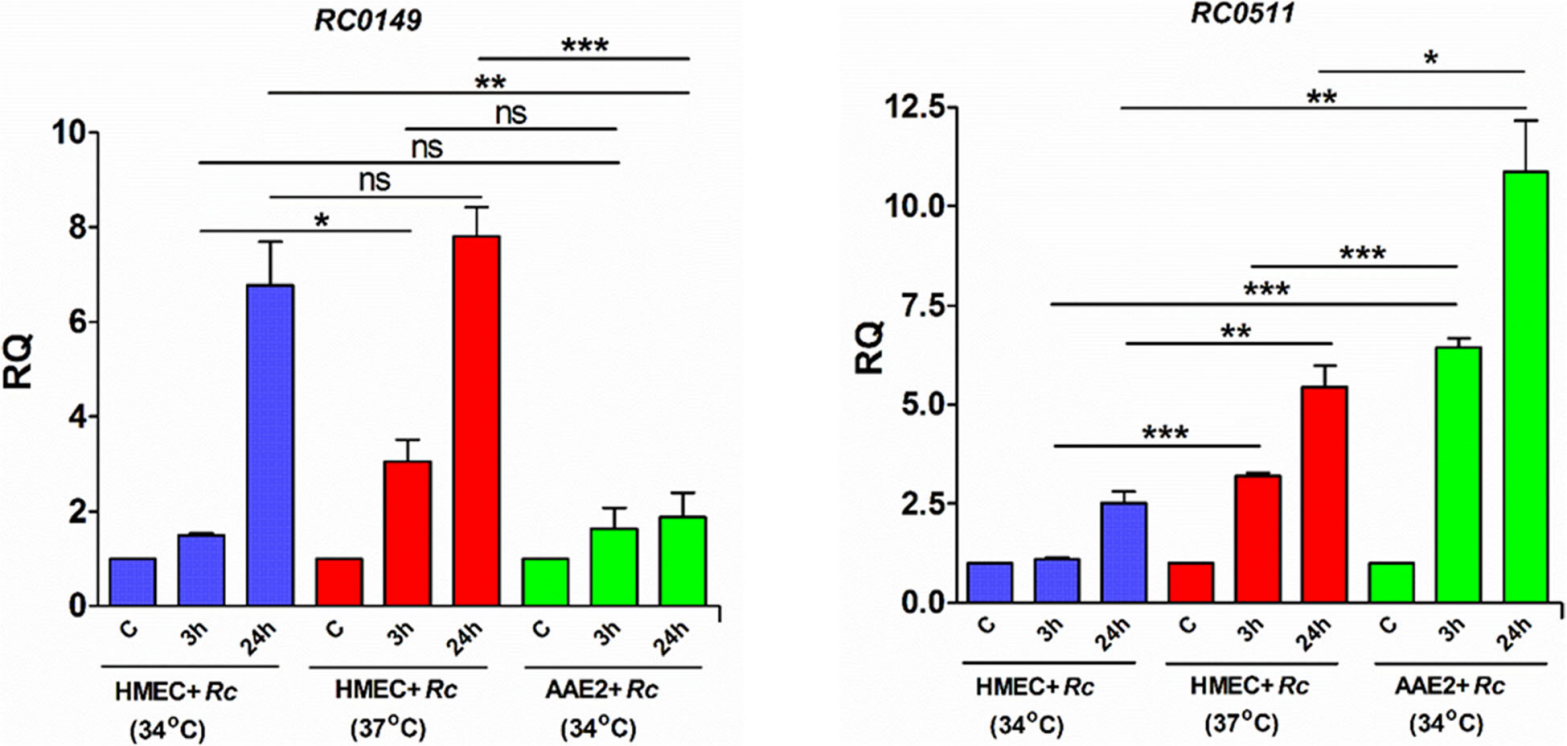
Quantitative PCR based validation of *R. conorii* coding genes differentially expressed during host and tick cell infection in vitro. *R. conorii* infected HMECs were maintained at either 37 °C or 34 °C, and AAE2 cells infected with *R. conorii* were maintained at 34 °C for the entire duration of the experiment. Total RNA was extracted at 15 min, 3 h and 24 h post infection, DNaseI treated, and reverse transcribed as described. Expression profile of *RC0149* and *RC0511* was quantified using gene-specific primers and 16S rRNA as endogenous control. Human and tick cells infected with *R. conorii* for 15 min served as baseline control and fold changes were calculated as described in methods. Data from three independent replicates is presented as mean ± SEM. ns = not significant, *p < 0.05, **p < 0.01, ***p < 0.001

To further confirm differential, host niche-dependent expression of *R. conorii* coding genes observed in our deep sequencing data, we selected *RC0511* and *RC0149* as the respective candidates differentially upregulated during the infection of tick and human cells (Tables 3 and 4). Our rationale for choosing these genes was based upon their protein function. The homologs of RC0511 are putatively annotated as transcriptional regulators belonging to the AbrB family, whereas RC0149 is an acetate kinase (EC 2.7.2.1) involved in the synthesis of acetyl-coA required for carbohydrate and lipid metabolism. Because previous studies have suggested an important role for the host temperature as a determinant of rickettsial gene transcription and tick cells are routinely maintained and infected at 34 °C, we infected host HMECs at both 34 °C and 37 °C to delineate the potential influence of temperature on gene expression. As expected, *RC0149* was significantly upregulated during the infection of HMECs when compared to AAE2 cells, and temperature (34 °C vs 37 °C) had no significant effect on the expression of this transcript. At 24 h post-infection, *RC0149* mRNA abundance displayed an average increase of 4-fold in HMECs (Fig. 1). On the other hand, expression of *RC0511* was slightly influenced by changes in temperature as indicated by higher expression (*p* < 0.05) in HMECs infected at 37 °C versus those at 34 °C. Finally, *RC0511* was expressed at much higher levels in tick cells at both 3 h and 24 h post-infection when compared to HMECs at the corresponding temperatures, thus suggesting contributory roles for both the host cell niche and its growth temperature in the differences in mRNA expression (Fig. 1).

### Identification of transcription start sites

**Fig. 2:**
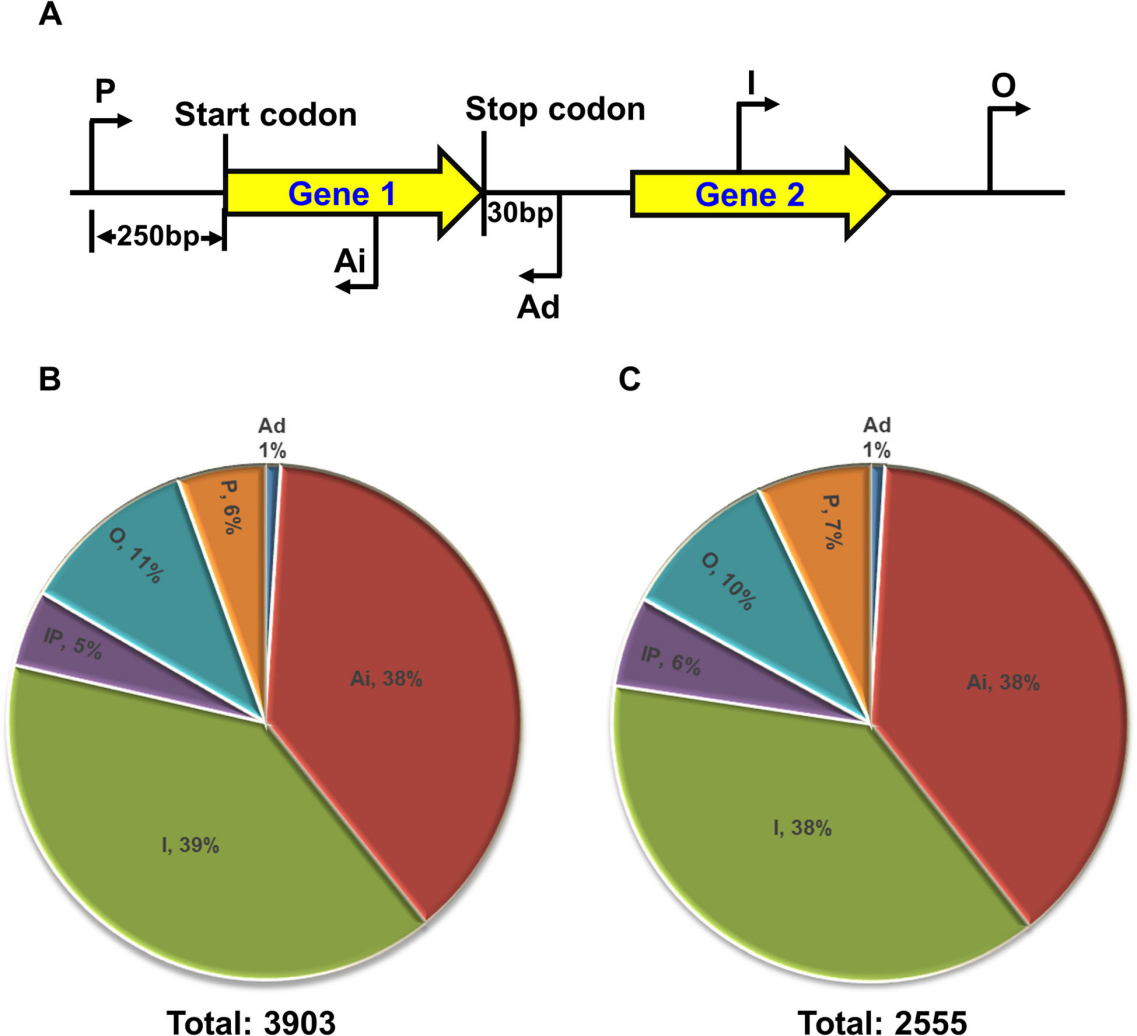
**a**: Identification and distribution of TSS in *R. conorii* during infection of host and tick cells, in vitro. **a**: Schematic showing the genomic location and classification of TSS. TSS were categorized as promoter (P), intergenic (I), orphan (O), antisense intergenic (Ai), and antisense downstream intergenic (Ad) based on their genomic location and distance from the translation start and stop positions of the respective gene. **b** and **c**: Distribution of different categories of TSS identified by TSSAR in *R. conorii* during the infection of tick AAE2 **b** and host HMECs **c**, in vitro. A total of 3903 and 2555 TSS were identified in *R. conorii* during infection of tick vector and human host cells, respectively. Majority of TSS (> 75%) were categorized as either intergenic or antisense intergenic depending on their genomic position

As rickettsial genomes harbor A-T rich polymorphic tracks resulting in the presence of spurious promoters and several genes are under the regulation from multiple promoters, we performed 5′-terminator exonuclease (TEX) treatment to enrich RNA for primary transcripts to identify transcription start sites (TSSs) in *R. conorii* genome. Sequencing following TEX treatment yielded an average of 82 and 85 million reads from infected tick cells and human ECs, respectively, of which 2.5 and 3.3 million reads mapped to *R. conorii*. The percentage of reads mapping to rickettsial genome is attributed to the limited efficiency of enrichment procedures, resulting in incomplete removal of eukaryotic host mitochondrial and noncoding RNAs lacking polyA tail, and bacterial ribosomal RNAs, and is in agreement with previous studies [23, 26]. Using TSSAR, we classified TSSs as promoter (pTSS), intergenic (iTSS), orphan (oTSS), antisense intergenic (AiTSS), or antisense downstream (AdTSS) based on the genomic location (Fig. 2a). A total of 3903 and 2555 TSSs were identified during in vitro infection of AAE2 and HMECs with *R. conorii* (Fig. 2b and c; Additional files 4 and 5). Of these, nearly 76% were classified as either intergenic or antisense intergenic and another 10–11% were categorized as ‘orphan’ TSSs depending on their genomic location (Fig. 2b and c). Our findings further revealed 214 and 181 pTSSs for coding transcripts in the tick vector and human host cells, respectively, of which 75 genes exhibited a difference in their TSS depending on the host niche (Additional file 6). The average length of 5’UTR was 80 and 71 bases for rickettsial coding transcripts in tick and human host cells, respectively. Of the 75 mRNA transcripts which exhibited a difference in their TSSs, *RC0368* (genomic location: 365526–366,383) exhibited the largest difference of 175 bases, yielding a longer transcript in tick cells (TSS: 366698) than in HMECs (TSS: 366523). Interestingly, RC1282 encoding for an adhesin Adr2 had a pTSS located 111 bases upstream of the translation start site during infection of AAE2 cells in comparison to only 12 bases upstream in HMECs (Additional file 6), indicating the use of alternative TSSs depending on the host cell. Collectively, our data reveal extensive antisense transcription in *R. conorii* irrespective of the host cell type, pTSSs for ~ 16% of the coding transcripts expressed in tick and human cells as the host, and existence of alternative pTSSs for several genes depending on the host niche.

### Identification of *R. conorii* sRNAs and riboswitches expressed during tick cell infection in vitro

**Fig. 3:**
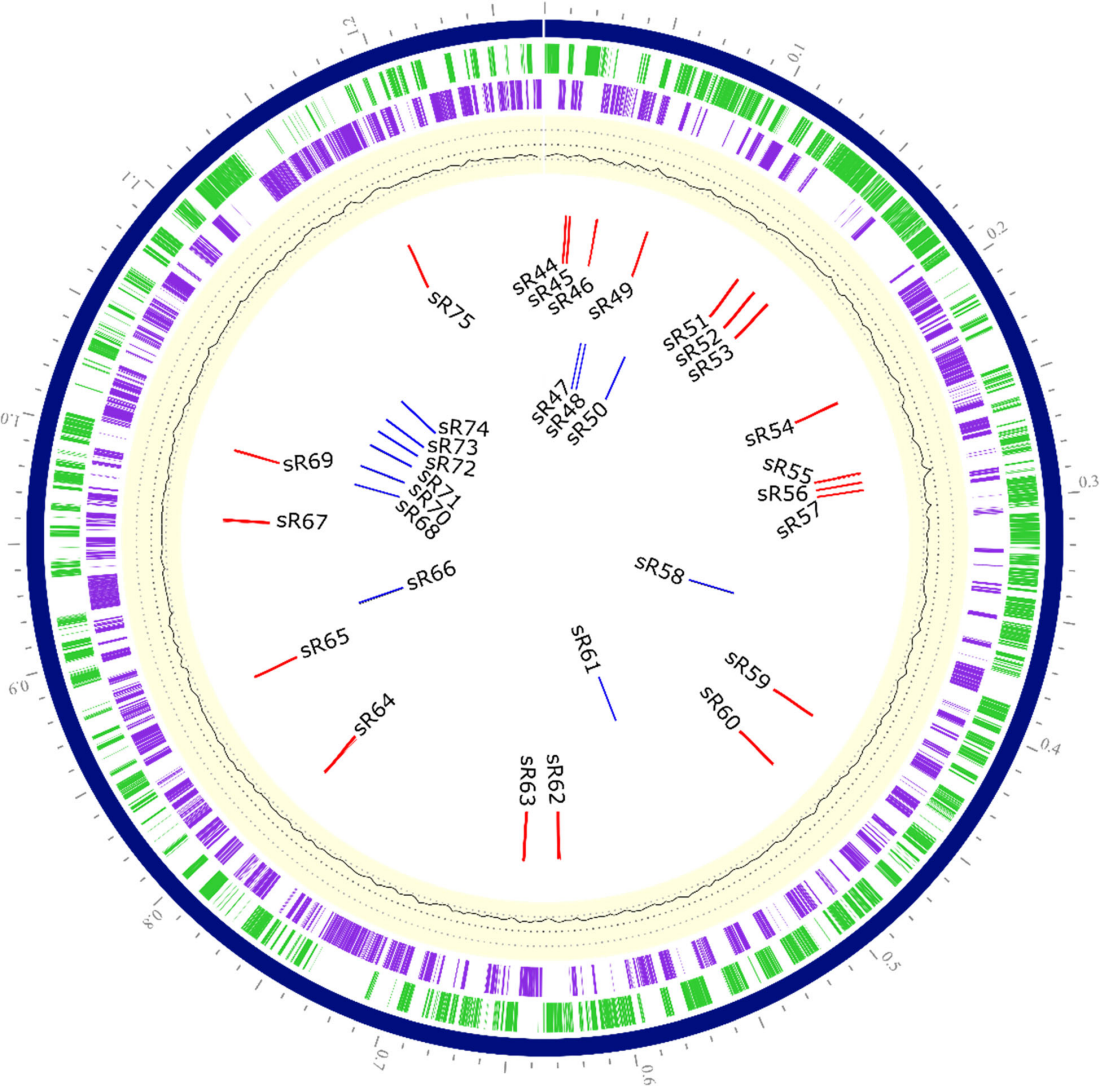
Circular chromosome map of *R. conorii* showing the genomic location of *Rc*_sRs identified in this study. The genome map of *R. conorii* was generated using PATRIC genome annotation (Genome ID: 27944.4) and different circles with bars represent: (1) Bright green (outermost): *R. conorii* coding genes annotated on the sense strand, (2) Purple: *R. conorii* coding genes annotated on the anti-sense strand, (3) Light yellow: GC skew, (4) Red bars: all cis-acting *Rc*_sR’s and riboswitch (*Rc*_sR59), and (5) Blue bars: all trans-acting *Rc*_sR’s identified in this study. The cis/trans acting sRNAs (*Rc*_sR45, sR46 and sR65) are also shown with red bars on second circle (inside to outside). *Rc*_sR1 through sR43 reported in our previous study [24] are not shown here

We have previously reported on the expression of small noncoding RNAs in *R. conorii* genome during in vitro infection of mammalian host cells [24]. To compare and contrast the noncoding landscape during infection of human host and tick vector cells, we performed deep sequencing of enriched bacterial transcriptome from HMECs and AAE2 cells infected with *R. conorii* for 24 h. The reads trimmed for base quality control were mapped to *R. conorii* genome (PATRIC annotation) for identification of sRNAs (both *cis-* and *trans-*acting) and riboswitches. In this study, we confirmed the expression of 43 *Rc*_sRs reported previously [24], and identified an additional 32 *Rc*_sRs found to be abundantly expressed in tick AAE2 cells (Fig. 3, Additional file 7). The expression of all of these sRNAs, including 16 *Rc*_sRs categorized as *cis*-acting (antisense of a coding gene) and 12 as *trans*-acting (intergenic) based on their location of origin (Additional file 7) were determined to be below the limit of detection in HMECs. Also, *Rc*_sR59 was identified as a riboswitch based on its location in the 5′-UTR region of RC0441, a hypothetical protein with considerable homology to the flagellar hook associated protein FlgK in *Bacillus* species (e-value 9e-06, 30% identity). In addition, three sRNA candidates (*Rc*_sR45, sR46, and sR62) were classified as both *cis-* and *trans-*acting owing to partial overlap with the neighboring (up or downstream) gene and the intergenic region, suggesting the possibility of regulating both the overlapping gene (antisense to sRNA) by direct base pairing and distant genes via partial base pairing. Further, 17 of the 32 *Rc*_sR’s were present on the leading strand and the remaining 15 originated from the lagging strand (Additional file 7). The average length of *Rc*_sR’s abundantly expressed during tick cell infection was 300 bases, with *Rc*_sR48 being the shortest (108 bases) and *Rc*_sR66 the longest (526 bases). Notably, *cis*-acting sRNAs were found on the anti-sense strands of several important genes encoding for outer membrane proteins (rOmpA, rOmpB and Sca4), an inner membrane protein of type IV secretion system (VirB6), protein translocase (SecF), proton/glutamate symporter (GltP), and prolipoprotein diacylglyceryl transferase (Igt) (Additional file 7). Thus, comparative deep sequencing enabled the identification of a number of new *Rc*_sR candidates abundantly expressed in tick host cells, implicating a role for their contributions to differential regulation of coding transcriptome during host-pathogen and vector-pathogen interactions.

### Validation of sRNA expression during *R. conorii* expression of host and tick cells

**Fig. 4:**
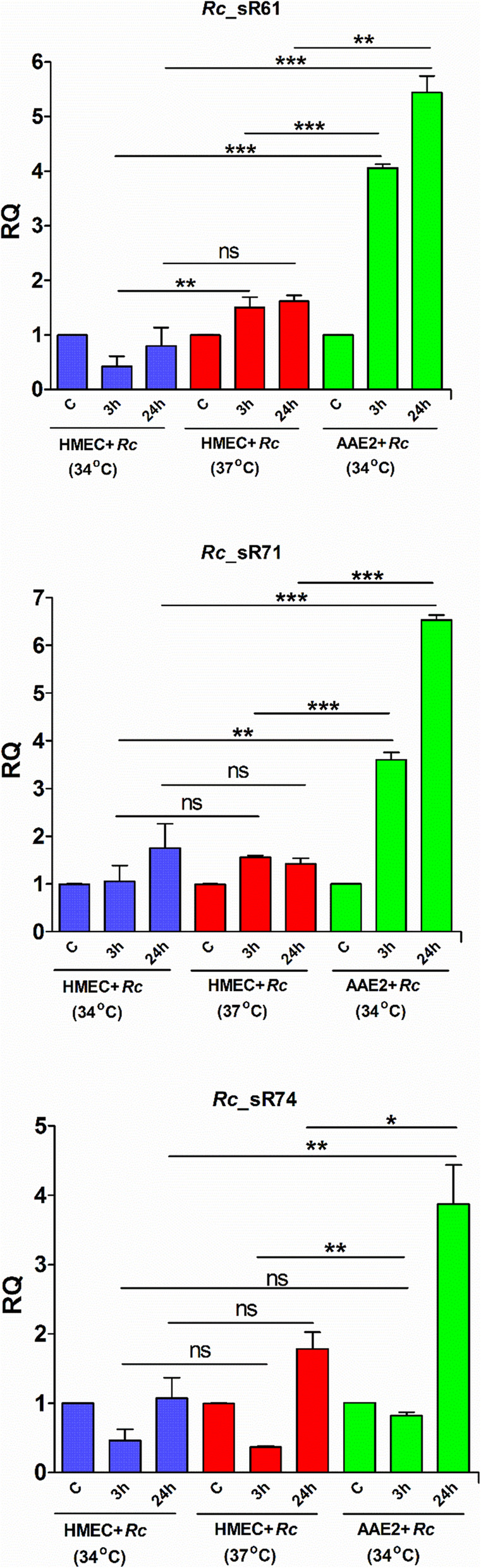
Quantitative PCR based validation of *R. conorii Rc*_sRs highly expressed during tick cell infection in vitro. *R. conorii* infected HMECs were maintained at either 37 °C or 34 °C, and AAE2 cells infected with *R. conorii* were maintained at 34 °C for the entire duration of the experiment. Total RNA was extracted at 15 min, 3 h and 24 h post infection, DNaseI treated and reverse transcribed as described. Expression profile of *Rc*_sR61, sR71 and sR74 which were highly expressed during tick cell infection and identified in our RNA seq data were quantified using sRNA-specific primers and 16S rRNA as housekeeping control. Human and tick cells infected with *R. conorii* for 15 min served as baseline control and fold changes were calculated as described in methods. Data from three independent replicates is presented as mean ± SEM. ns = not significant, *p < 0.05, **p < 0.01, ***p < 0.001

To further validate *Rc*_sR’s identified to be expressed during *R. conorii* infection of tick cells, expression of three trans-acting sRNAs (*Rc*_sR61, sR71, and sR74) was assessed by quantitative RT-PCR using sRNA specific primers and 16S rRNA as an endogenous control. For comparative analysis, *Rc*_sR expression was measured at 3 h and 24 h post-infection in HMECs grown and maintained at 34 °C and 37 °C, and in AAE2 cells maintained at 34 °C. The temperature had no influence on the transcript abundance of *Rc*_sR71 and *Rc*_sR74, but *Rc*_sR61 displayed a statistically significant difference in its expression at 3 h in HMECs infected at 34 °C versus 37 °C (Fig. 4). All three sRNAs tested were highly upregulated at both 3 h and 24 h post-infection during *R. conorii* infection of AAE2 cells when compared to HMECs as the host cells. At 24 h post-infection, expression levels in tick cells were higher by an average of 3–5 fold than those seen in HMECs, corroborating our findings from the deep sequencing approach (Fig. 4).

### Comparative analysis of 6S RNA expression during HMEC and AAE2 infection

**Fig. 5:**
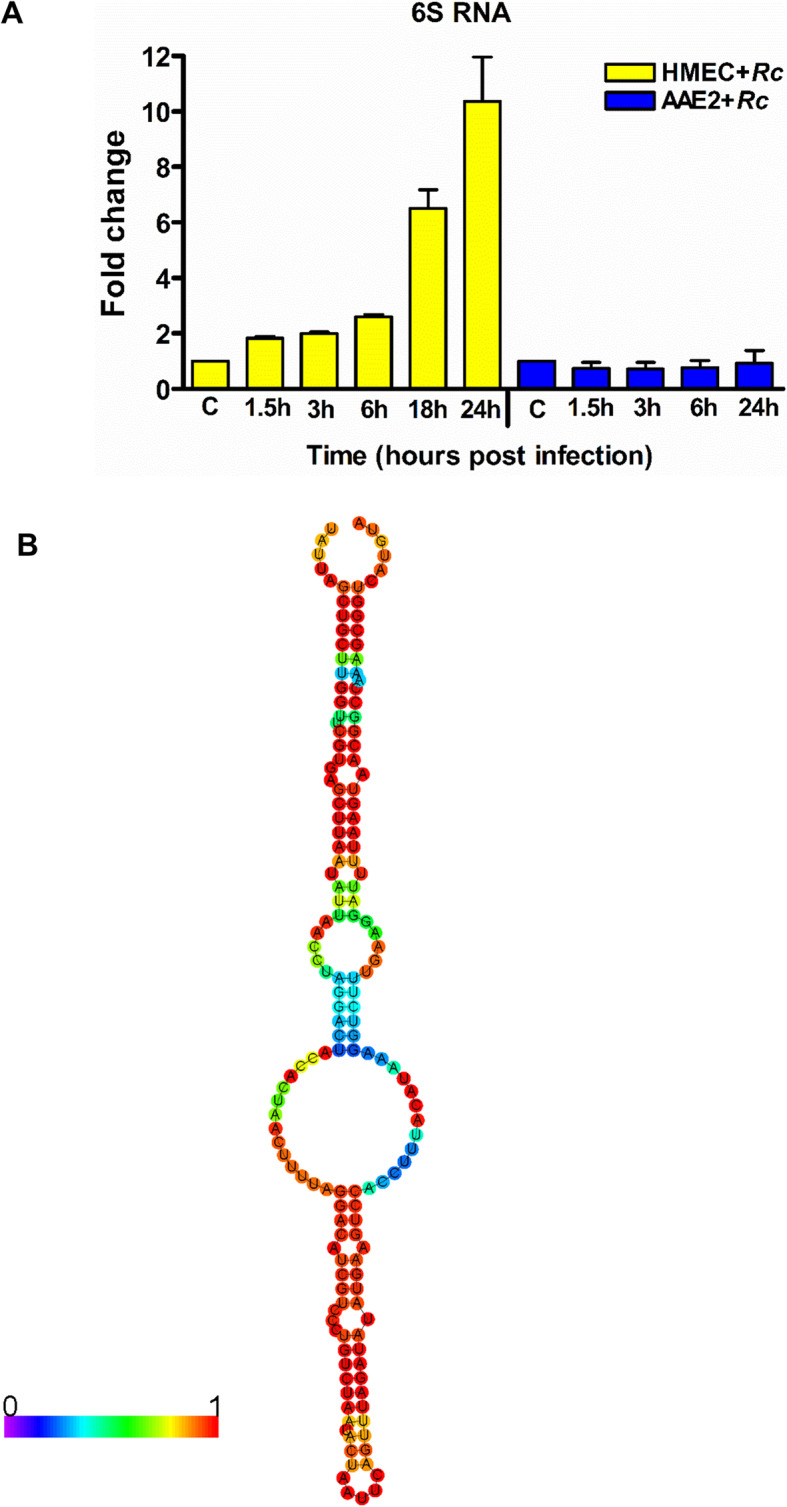
**a**: Expression profile and secondary structure of *R. conorii* 6S RNA. A. Expression of 6S RNA during the infection of HMECs and tick cells in vitro. HMECs and AAE2 cells were infected with *R. conorii* (MOI = 50) and total RNA was extracted at different time interval between 0.5 to 24 h post infection. Total RNA was DNaseI treated, reverse transcribed, and expression of 6S RNA was assessed by Taqman based quantitative PCR using 6S RNA specific primers and probe, and 16S rRNA as internal control. The data was calculated using expression at 0.5 h as baseline control. 6S RNA was significantly upregulated (p < 0.05) at all time point during the infection of HMECs, while steady state expression with no significant change was observed during tick cell infection. Data from three independent replicates is presented as mean ± SEM. **b**. Predicted secondary structure of 6S RNA. The secondary structure of *R. conorii* 6S RNA predicted by RNAfold web server (University of Vienna) resembles the conserved structure observed for most bacterial 6S RNAs. The color coding on bases indicate base pairing probability of 0 to 1 (purple to red)

6S RNA (*ssrS*) is a small RNA regulator of RNA polymerase highly conserved in most bacteria, including all *Rickettsia* species. We have previously reported on the presence of *R. conorii* 6S RNA (*Rc*_sR36) transcripts during the infection of HMECs and confirmed its expression by Northern blotting [24]. To further compare and quantify its expression during in vitro infection of mammalian host vis-à-vis tick cells, we performed a Taqman based RT-qPCR assay on total RNA extracted at different times ranging between 15 min and 24 h post-infection. Owing to the obligate intracellular lifestyle of *Rickettsia* species, the earliest time point of 15 min was used as the baseline control. 6S RNA was significantly upregulated at all times compared to its basal level of expression (at 15 min) in infected HMECs, with the highest increase of ~ 10-fold at 24 h (Fig. 5a). In sharp contrast, 6S RNA expression in tick cells remained unchanged in comparison to the baseline control at different times post-infection, suggesting differential regulation during host-pathogen and vector-pathogen interactions, in vitro (Fig. 5a). The secondary structure of *R. conorii* 6S RNA (*Rc*6S), as determined by RNA-fold, resembled that of *E. coli* 6S RNA (*Ec*6S). Similar to *Ec*6S, *Rc*6S RNA also displays a central bulge of single stranded nucleotides critical for the binding to sigma 70 transcription factor and forms a double stranded stem like structure with minor bulges (Fig. 5b).

## Discussion

In this study, we have analyzed the coding and non-coding transcriptomes of *R. conorii* during host-pathogen and vector-pathogen interactions, in vitro. Using a high throughput RNA sequencing approach, we have identified differentially expressed genes depending on the host niche and 32 novel *R. conorii* sRNAs abundantly expressed during tick cell infection as compared to host HMECs. Additionally, we performed enrichment of primary transcripts using 5′ terminator exonuclease to determine the transcription start sites for 214 and 181 *R. conorii* genes expressed during infection of vector and human host cells, respectively.

As obligate intracellular pathogens, a majority of spotted fever group rickettsiae are transmitted to humans via a tick bite. Although some *Rickettsia* species, example *R. rickettsii*, are detrimental to ticks infected by transovarial transmission in nature, a majority are known to persist and survive in infected vectors and lead to serious disease in humans [27]. However, the mechanisms by which pathogenic *Rickettsia* species adapt to different host environments remain obscure. Our findings reveal that while the same core set of genes are abundantly expressed during the infection of both cell types (Tables 1 and 2), a greater number of other *R. conorii* genes are transcribed above the limit of detection and differentially expressed during the infection of tick vector cells in direct comparison to human endothelial cells, in vitro (Additional file 2). Interestingly, *RC0497* was determined to be the most abundantly expressed gene in both cell types (Tables 1 and 2). Recently, we have characterized RC0497 as an ampD domain containing N-acteylmuramoyl-L-alanine amidase involved in peptidoglycan hydrolysis and demonstrated its localization on the septal regions in dividing bacteria and on the membranes of vesicles protruding from the rickettsial cell wall [28]. A simultaneous study has further revealed that RC0497 is also secreted into the culture supernatants during infection of endothelial cells in vitro and is readily detectable in the serum of infected patients, projecting it as a promising candidate for the design and development of rapid diagnostics [29]. Additionally, heat shock chaperone (*groEL*), cold shock protein of CSP family (*cspA*), CarD like transcriptional regulator, and anti-toxins (*vapB* and anti-toxin of *relE*) were ubiquitously expressed in both infected HMECs and tick cells. Previous studies have reported constitutive expression of rickettsial *groEL* during active growth conditions (mid-log phase)*,* down-regulation during slow growth and starvation, and up-regulation during heat shock [5, 30, 31]. The *groEL* is an essential molecular chaperone required for proper folding of proteins. In endosymbiotic bacteria, *groEL* is presumed to play a vital role in restoring bacterial fitness, which is compromised due to the accumulation of mutations arising from the bottlenecks experienced during transovarial transmission [32–34]. On the other hand, proteins belonging to the CSP family are activated during oxidative, osmotic, as well as cold stress conditions, and required for bacterial adaptation and intracellular survival [35–37]. For instance, deletion of CSP family proteins in *Listeria monocytogenes* results in increased susceptibility to oxidative stress and impaired host cell invasion and intracellular growth [38]. Also, *cspA*-lacking mutants of *Brucella* display differential expression of 446 genes involved in energy metabolism and the biosynthesis of amino- and fatty acids. Notably, genes involved in type IV secretion system are also downregulated, indicating its role in virulence, metabolism, and adaptations to host microenvironment [39, 40].

Spotted fever group rickettsiae employ actin-based motility for intracellular movements and intercellular spread. In this regard, bacterial RickA localized at the pole has been implicated in the activation of host Arp2/3 complex and formation of actin tails for dissemination during early stages of infection [41]. Thus, our finding of abundant expression of *rickA* irrespective of the host cell type is not surprising. Earlier studies on *R. parkeri rickA* and *sca2* deletion mutants have documented their ability to spread to all organs of *A. maculatum*, but exhibit significantly lower rickettsial burden in comparison to the wildtype, indicating that these genes are necessary for efficient dissemination to different tissues of the host [42]. At least five bicistronic modules coding for a stable toxin and a liable antitoxin have been reported in most rickettsial genomes. Among them, VapC toxin secreted into the cell cytosol and exhibiting RNase activity is presumably involved in mediating the deleterious effect of ciprofloxacin during the infection of host cells, in vitro [43, 44]. Furthermore, a role for the *relBE* module in tolerance of *E. coli* to antibiotics has also been suggested [45, 46]. It is, therefore, possible that *Rickettsia* ubiquitously express these genes irrespective of the host cell type to facilitate their spread, persistence, and tolerance of host and environmental stress responses.

We identified 132 and 305 differentially upregulated genes (log_2_ fold change ≥2.0) in human ECs and tick AAE2 cells, respectively. Of these, nearly 49% (61 of 125) encoding for hypothetical proteins in *R. conorii* were uniquely expressed in tick cells (Additional file 2). Consistent with this finding, *R. rickettsii* transcripts for numerous hypothetical proteins putatively encoding for iron permease, thioredoxin, and ankyrin repeat proteins undergo differential modulation due to temperature changes and blood feeding in tick vectors [7, 8]. In further agreement with earlier reports showing increased expression of type IV secretion components and putative effectors of *R. rickettsii* in tick vectors during blood meal [8], we also observed up-regulation of transcripts encoding for VirB6, VirB8, and VirB9, ankyrin repeat proteins (Ank proteins), tetratricopeptide repeat proteins (TPR), acid phosphatase, and metalloprotease during tick cell infection (Additional file 2). Evidence suggests that metalloproteases and Ank proteins of several bacteria, including *Rickettsia* species, are secreted into extracellular milieu, interact with host components, and regulate host immune responses [47, 48]. As a predicted cysteine protease secreted via type IV secretion system, RARP-2 (rickettsial ankyrin repeat protein-2) has recently been documented for its involvement in the fragmentation of trans-Golgi network, resulting in the disruption of protein trafficking to plasma membrane [49]. *Orientia* Ank proteins are known to modulate NF-κB transcriptional activation, protein secretion, endoplasmic reticulum (ER) stress, SCF1 ubiquitin ligase assembly, and Golgi to ER retrograde trafficking, thus impacting the replication and/or pathogenesis in the host cell [50–52]. Similarly, metalloproteases produced by several bacteria contribute to a wide array of pathomechanisms such as hemorrhagic tissue damage, increased vascular permeability, degradation of proteins and peptides for bacterial nutrition, and adhesion to and invasion into host cells [53]. For example, *Serratia grimesii* metalloprotease grimelysin is secreted through outer membrane vesicles, hydrolyzes actin, and aids in bacterial invasion of host cells [54]. The *E. coli* SslE, a zinc metalloprotease with mucinase activity, facilitates penetration of mucus layer and is involved in adhesion of bacteria to host cell [55]. TPR containing proteins in bacterial pathogens function as determinants of virulence, host cell adhesion and intracellular survival, inhibition of phagosomal maturation, transduction of stress signals, and chaperone activity [56]. For instance, *Pseudomonas aeruginosa* PcrH, a TPR domain containing protein, functions as a class II chaperone and facilitates stabilization of translocators (PopB and PopD) essential for the translocation of toxins into host cytosol, and protein kinase G (PknG) of *Mycobacterium tuberculosis* secreted into macrophages inhibits phagosome-lysosome fusion to ensure intracellular survival and bacterial replication [57, 58]. Further, acid phosphatases from *Francisella tularensis* secreted into host cytoplasm also function as virulence factors involved in the inactivation of NADPH oxidase and inhibition of oxidative burst in host macrophages [59]. Thus, upregulation of genes coding for TPRs, metalloproteases, and acid phosphatases in infected tick cells may modulate host responses (example, prevention of oxidative burst) to facilitate rickettsial colonization and persistence in arthropod vectors. Since previous studies have demonstrated considerable differences in rickettsial gene expression between controlled (in vitro) and natural (in vivo) conditions as a consequence of temperature shift and a majority of *R. rickettsii* genes are differentially modulated by feeding, target tissue (salivary glands or midgut), and gender of the tick vector [5, 7, 8], studies to further comprehend the roles of these proteins in tick vectors during natural transmission will provide a better understanding of the rickettsial adaptation mechanisms during host-pathogen and vector-pathogen interactions. An important consideration in this regard is that acquisition of bacteria by tick vectors during natural blood feeding may vary greatly and environmental stimuli may also have a profound impact on pathogen intake, maintenance and transmission as part of the natural life-cycle.

We employed the standard approach of 5′-terminator exonuclease treatment to enrich primary transcripts and to identify *R. conorii* transcription start sites during the infection of human ECs and tick vector cells. Regardless of the host cell type (HMECs or AAE2 cells), nearly 76% of the total TSSs identified in *R. conorii* are either categorized as antisense or internal based on their genomic origin (Fig. 2b and c; Additional files 4 and 5). The existence of a large proportion of antisense transcription in organisms belonging to archaea, prokaryotes, and eukaryotes is now well appreciated [60–62]. For example, differential RNA sequencing of *E. coli* grown in three different conditions has led to the identification of a total of 14,868 TSSs, of which nearly 74% correspond to either potential antisense RNAs or are internal to annotated genes [63]. Genome wide mapping of TSSs in *Leptospira interrogans* has also identified more than 2800 TSSs, of which 12% are classified as antisense TSS and 53% designated as internal [64]. Similarly, 13 and 63% of the total 6042 TSSs identified in *Borrelia burgdorferi* during the infection of mammalian host have also been classified as antisense and internal TSSs, respectively [65]. Of the 1576 annotated ORFs in *Helicobacter pylori*, 46% (721) of the genes contain at least one antisense TSS and nearly 17% of the 2496 TSSs are categorized as intergenic based on their genomic location [66]. Although these and our current findings reveal the prevalence of antisense transcription in most organisms including *R. conorii*, the biogenesis and roles of antisense transcripts in the regulation of the coding transcriptome is not yet clear. Antisense transcription is generally considered a ‘biological noise’ originating due to inefficient transcriptional termination by Rho-independent terminators and the presence of spurious promoters arising from point mutations, especially in AT rich genomes like that of *Rickettsia* species [67]. Nevertheless, antisense transcripts are known to act as genetic switches controlling bacterial competence, virulence, and regulation of toxins [68–70]. Additionally, several antisense RNAs are known to function as post-transcriptional inducers or inhibitors of gene expression and protein translation, and as regulators of plasmid copy numbers through inhibition of primer maturation, thus impacting several cellular functions, including biofilm formation, quorum sensing, and toxin synthesis [71]. For example, while cis-encoded antisense RNA of *mucD* (mucD_AS) regulates *mucD* expression and induces biofilm formation in *Pseudomonas aeruginosa, micF * as an asRNA in *E. coli* inhibits OmpF by destabilizing the mRNA and inhibiting translation [72, 73]. It is now also evident that several antisense promoters in *E. coli* are functional and involved in the fine tuning of gene expression [74]. Thus, it is likely that although antisense transcription in bacterial genomes is pervasive, some of these transcripts encode for a bona fide function and play a vital role in the survival, fitness, and pathogenesis of the organism.

We have identified primary TSSs for 16% of the genes, of which 75 *R. conorii* coding transcripts exhibit difference in their pTSS during in vitro infection of human host and tick vector cells (Additional file 6). The occurrence of secondary TSS (sTSS) is also common in most bacterial and archaeal genomes. For example, of the 14,868 TSSs in *E. coli*, 1707 and 850 have been classified as primary and secondary TSSs, respectively [63]. Similarly, ~ 2300 and ~ 3100 TSS are categorized as sTSS in *Leptospira* grown at 30 °C and 37 °C, respectively, indicating a role for environmental cues in the determination of transcription start sites [64]. In *R. prowazekii*, the citrate synthase gene is under the control of two promoters and our recent transcriptomic analysis revealed that 18 genes exhibit differences in their TSSs during rickettsial infection of HMECs and AAE2 cells [25, 75]. Consistent with our previous report for *R. prowazekii*, the coding transcripts expressed in *R. conorii* during the infection of tick vector cells were longer than those in human host cells. It is thus plausible that the length of 5′-UTR may influence the half-life of mRNA and have an impact on translational efficiency. Further studies using reporter constructs are likely to shed light on the roles of alternative/secondary TSSs of rickettsial transcripts in transcriptional regulation during rickettsial persistence and pathogenesis in the tick vector and human host, respectively.

One of the key findings in this study is the identification of 31 novel cis- or trans-acting *Rc*_sR’s and one riboswitch abundantly expressed in *R. conorii* during the infection of tick vector cells. Of these, expression of three trans-acting *Rc*_sR’s [sR61, sR71 and sR74] was further confirmed by quantitative RT-PCR (Figs. 3 and 4, Additional file 7). These results are in congruence with the published literature reporting exclusive expression of selective bacterial small RNAs in response to external stimuli, such as stress, nutrient starvation, temperature shift, and host environment [18, 19, 21]. In *R. prowazekii*, 67 cis-acting and 26 trans-acting sRNAs were abundantly expressed only during the infection of tick AAE2 cells [25]. *Buchnera* is an obligate nutritional endosymbiont maintained by transovarial transmission in aphids. This bacterium is shown to express 26% of its sRNA repertoire based on the life stage of the aphid host. Furthermore, 21% of *Buchnera* sRNAs are expressed depending on the host plant, indicating the bacterial potential to alter its transcriptome based on the availability of nutrients from the aphid host [76, 77]. In *Borrelia burgdorferi*, nearly 43% of sRNAs are demonstrated to be temperature dependent, of which 128 and 303 sRNAs are upregulated at 23 °C (ambient temperature of tick vector) and 37 °C (temperature of the human host), respectively. Interestingly, two antisense sRNAs regulating *bba66*, a gene required for eukaryotic host infection through tick transmission, are upregulated at 37 °C, indicating a potential role for these sRNAs in the regulation of coding transcriptome during host-pathogen interactions [18]. Eighty-four sRNAs in *E. coli* are differentially expressed solely during fermentation, yet 139 sRNAs involved in biofilm formation, motility, regulation of outer membrane proteins, and maintenance of cell envelope are significantly up- or down-regulated during chemical stress [78]. Therefore, it is likely that 32 *Rc*_sR’s identified in this study, play an important role in regulating rickettsial transcriptome during vector-pathogen interactions. Despite the absence of hfq, an RNA chaperone involved in sRNA-mRNA binding, in rickettsial genomes, we have previously confirmed the interactions between *Rc*_sR42 and *cydA* mRNA in vitro, suggesting the possibility of direct binding as a mechanism of action by rickettsial sRNAs [24]. Further studies focused on the identification and functional characterization of target genes regulated by these sRNAs will shed light on the roles of *R. conorii* sRNAome in host-pathogen-vector interplay.

Another striking and intriguing observation of this study is the differential expression of 6S RNA during the infection of human versus tick cells. While 6S RNA (*ssrS*) expression steadily increased in HMECs, no significant differences compared to the basal levels were observed during the infection of tick cells (Fig. 5). It is now increasingly evident that 6S RNA in a number of bacteria is differentially expressed depending on the growth stage. For instance, *E. coli* and *Legionella* 6S RNA accumulates at ~ 10 fold higher levels during stationary growth phase, while the *Bacillus* 6S RNA expression changes only 2–3 fold between the exponential and stationary phases [79]. Similarly, 6S RNA expression in *R. prowazekii* doubles at 48-72 h post-infection in comparison to early stages (1.5 to 3 h), during the infection of host endothelial cells, in vitro [22]. The *Coxiella* 6S RNA also shows the highest accumulation in its small cell variant form at 14 days post-infection [80]. However, *Wolbachia* 6S RNA accumulates at higher levels during fast replication and infection of germ line cells, compared to stationary growth and infection of somatic cells, respectively [81]. In *Borrelia*, 6S RNA exhibits highest expression levels in *Ixodes* unfed nymphs compared to fed nymphs and *ssrS* deletion mutant is compromised in infectivity of mice. Interestingly, despite being seropositive, the number of antigenic proteins reacting with murine immune system are considerably less in the ∆*ssrS* mutant compared to wild type or *ssrS* complemented strain, implicating a role for 6S RNA in the regulation of the expression of genes targeted by the murine adaptive immune system [82].

**Fig. 6:**
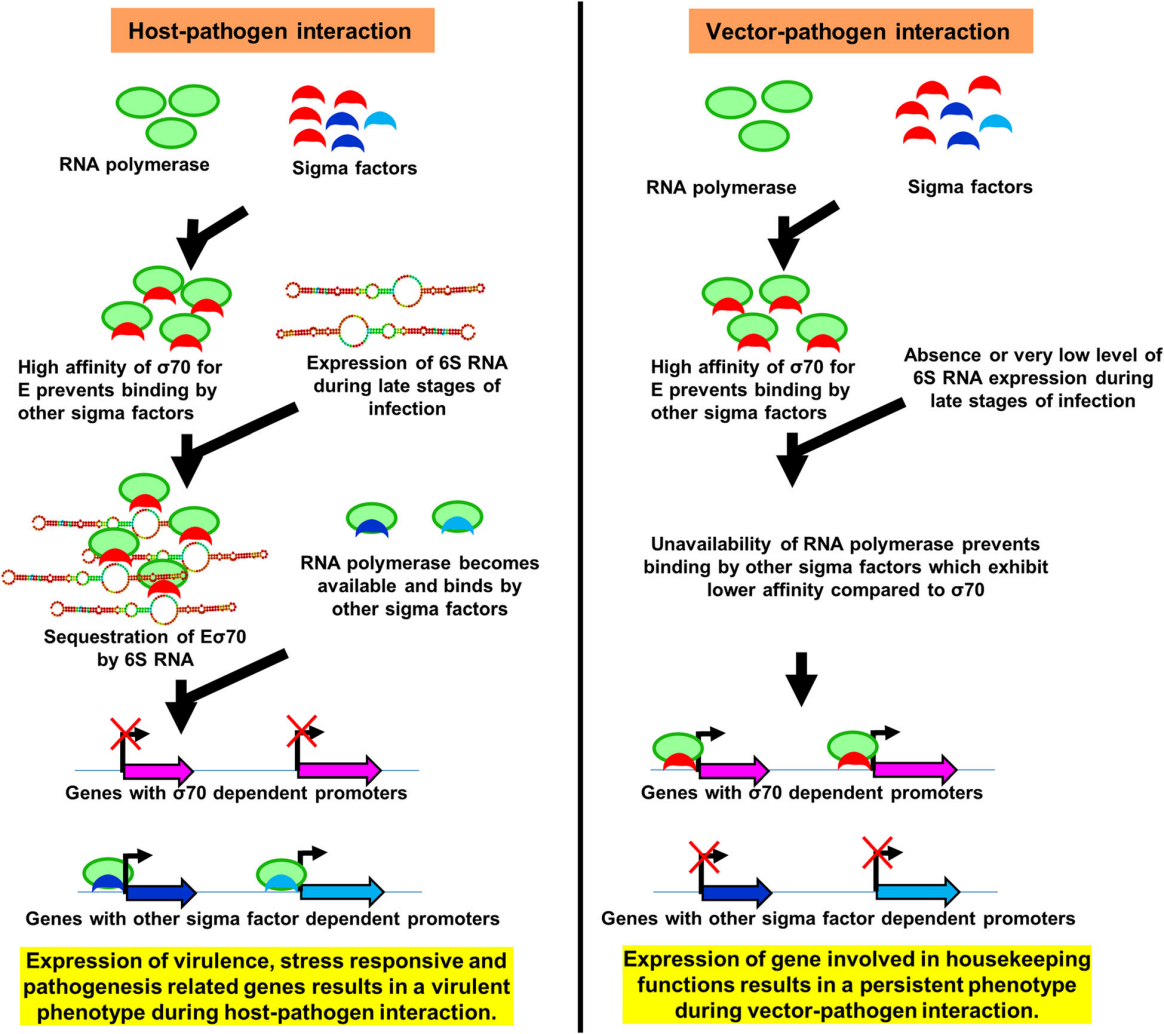
Proposed model of 6S RNA based regulation of *R. conorii* coding transcriptome during host-pathogen and vector-pathogen interactions

Functional and crystallographic studies have shown 6S RNA to be a global transcriptional regulator, which tightly binds to housekeeping holoenzyme Eσ^70^ with high specificity and is involved in the regulation of transcription of genes with σ^70^ dependent promoters. Although initial studies showed downregulation of genes containing σ^70^ promoters due to increased expression of 6S RNA, later findings revealed that accumulation of 6S RNA can result in both up and downregulation of several genes in a promoter-specific manner [79]. The deletion of *E. coli* 6S RNA resulted in increased expression of genes under Eσ^70^ promoters, while genes regulated by Eσ^38^ promoters were downregulated [83]. Under normal growth conditions, the cellular concentration of sigma factors exceeds RNA polymerase (E) concentration resulting in increased competition among the sigma factors to bind to E, and most abundantly expressed σ^70^ exhibiting higher affinity (*K*_d_ = 0.26 nM) for E can actively prevent binding of other sigma factors exhibiting low affinity (example: σ^38^, *K*_d_ = 4.26 nM) to compete for RNA polymerase. Thus, increased expression of 6S RNA can sequester Eσ^70^ allowing other sigma factors including σ^38^ to compete more effectively for binding to E, thus allowing for increased expression for genes regulated by these sigma factors [84]. Though *E. coli* encodes for seven sigma factors regulating proteins involved in housekeeping, nitrogen metabolism, heat shock, iron transport, flagellar proteins, and several other cellular functions during stationary phase growth, *R. conorii* genome harbors only two conserved sigma factors, namely RpoD (σ^70^) and RpoH (σ^32^) involved in regulating housekeeping and heat shock proteins, respectively. In addition, the existence of extracytoplasmic functional sigma factors (ECFs), small regulatory proteins exhibiting divergent sequences relative to known sigma factors, is well documented in many bacterial genomes and nearly 2700 ECFs from hundreds of bacterial genomes have been reported to date [85, 86]. Upon receiving a stimulus, ECFs are synthesized and released, which then bind to E and regulate a wide array of genes involved in oxidative stress, resistance to high temperatures and antibiotics, starvation responses and other cellular functions. For instance, the ECF RpoE4 of *Rhizobium etli* is known to regulate 98 genes, a majority of which are involved in cell envelope biogenesis and stress responses [87]. Rickettsial genomes also encode for several hypothetical proteins and it is possible that some of these proteins might act as ECFs regulating transcriptional expression of genes involved in multiple pathways. Collectively, based on these reports and our findings in this study of the increased expression of 6S RNA during infection of HMECs but not tick cells, differential expression of *R. conorii* genes depending on the host niche, and identification of a significantly greater number of genes to be transcribed during the infection of tick cells than in host HMECs, we propose a model (Fig. 6) implicating a role for 6S RNA in the regulation of *R. conorii* coding transcriptome during host-pathogen and vector-pathogen interactions. Based on our model, it is likely that increased accumulation of 6S RNA at later stages (18-24 h) of infection of the host cells results in the sequestration of Eσ^70^, leading to the downregulation of housekeeping genes, thus allowing other sigma factors including ECFs to actively bind to RNA polymerase and regulate the expression of genes involved in functions such as oxidative stress response leading to virulence phenotype during host-pathogen interactions. In contrast, lower levels with no detectable changes in the expression profile of 6S throughout the course of *R. conorii* infection of tick cells directly results in reduced sequestration and increased availability of Eσ^70^, resulting in the transcription of more housekeeping genes and leading to a persistent phenotype during vector-pathogen interactions. Further ongoing investigations focused on characterizing the roles of 6S RNA in rickettsial genomes will shed light on the regulatory mechanisms of this global transcriptional regulator during tripartite host-pathogen-vector interactions.

## Conclusions

*R. conorii* is an arthropod vector-borne obligately intracellular pathogen, which survives in its arthropod host as part of the natural life-cycle, but causes human disease leading to vascular edema, infection of central nervous system and other organs, and mortality if not diagnosed and treated early. However, the mechanisms by which *Rickettsia* species regulate their transcriptome during persistence (vector-pathogen interaction) and pathogenesis (host-pathogen interaction) is not clearly understood. In this study, we decoded the coding and non-coding transcriptional landscape and identified transcription start sites of coding transcripts of *R. conorii* during in vitro infection of human host and tick vector cells. Our results suggest a greater number of *R. conorii* genes to be transcribed above the limit of detection during the infection of tick cells, of which 305 genes are differentially upregulated (> 2-fold) when compared to human host cells. In contrast, only 132 *R. conorii* genes are upregulated at > 2 fold in HMECs when compared to tick cells. Enrichment of primary transcripts by Terminator 5′-Phosphate-dependent Exonuclease treatment enabled the identification of 3903 and 2555 TSSs in *R. conorii* during the infection to tick and human host cells, respectively. Most strikingly, we have further identified 32 novel *Rc*_sRs, which are highly expressed in *R. conorii* during the infection of tick vector cells. Finally, 6S RNA was identified to be differentially expressed during host-pathogen and vector-pathogen interactions, implicating a role for this common bacterial noncoding RNA in the regulation of rickettsial transcriptome depending on the host niche.

## Methods

### Cell culture

Human microvascular endothelial cells (HMECs), an immortalized cell line of dermal origin, were obtained from the Centers for Disease Control, Atlanta, GA. HMECs were grown in MCDB131 medium supplemented with 10% v/v fetal bovine serum (FBS) (Aleken Biologicals), 10 ng/mL epidermal growth factor (Thermo Fisher Scientific), 10 mM L-glutamine (Thermo Fisher Scientific), and 1 μg/mL hydrocortisone (Sigma) in a cell culture incubator maintained at 37 °C and 5% CO_2_. Cells from *Amblyomma americanum* ticks (AAE2) were kindly provided by Dr. Ulrike Munderloh (University of Minnesota, USA). The AAE2 cells were cultured in L15B complete medium supplemented with 20% v/v FBS (Harlan Bioproducts) and maintained at 34 °C as described previously [88]. The Vero E6 (African green monkey kidney fibroblasts) cells were cultured in Dulbecco’s modified eagle medium (DMEM) supplemented with 2–10% v/v FBS at 37 °C in an atmosphere of 5% CO_2_ [89]. All cell lines were exempt by the University of Texas Medical Branch (UTMB) Institutional Review Board (IRB), and approved by the UTMB Institutional Biosafety Committee (IBC) for the use in these studies.

### Preparation and quantification of *R. conorii* stocks

Stocks of *Rickettsia conorii* were prepared in Vero E6 cells using established protocols and procedures [90]. *R. conorii* stocks prepared from the yolk-sacs of fertilized eggs as described earlier [91] were used for further propagation in Vero cells. Briefly, confluent Vero cell monolayers in DMEM containing 2% FBS were infected with *R. conorii* and incubated at 35 °C, 5% CO_2_. The cultures were monitored microscopically at about 24 h intervals and *R. conorii* was harvested when approximately 30–40% of Vero cells detached from the culture surface. Rickettsiae were purified by differential centrifugation, enumerated by quantitative PCR using primers specific for rickettsial citrate synthase (*gltA*) gene and by plaque assay as described earlier, and stored as ≤500 μl aliquots at -80 °C until further use to prevent repeated freezing and thawing [24].

The growth of *R. conorii* in human and tick cells was determined by quantitative PCR as described earlier [24, 92]. Briefly, HMECs infected with *R. conorii* were maintained at 34 °C or 37 °C, while AAE2 cells were infected at 34 °C for the duration of the experiment. At 24 h post-infection, cell monolayer was washed twice with sterile PBS and incubated with DNase I (10 U/ml for 30 min) to remove extracellular bacteria. At the end of incubation, monolayer was washed twice with sterile PBS and genomic DNA was extracted using a DNeasy blood and tissue kit (Qiagen) following the manufacturer’s protocol. Absolute quantification of rickettsial load was performed by q-PCR using citrate synthase gene specific primers described previously [92, 93].

### Infection of HMECs and AAE2 cells with *R. conorii* and total RNA extraction

Monolayers of HMECs at about 80 to 90% confluence were infected with *R. conorii* at an MOI of 50 following standard protocols. The MOI of 50 was chosen to increase the abundance of bacterial transcripts as eukaryotic transcripts, despite enrichment for microbial coding and non-coding RNAs, tend to interfere with library preparation and sequencing [23]. *R. conorii* infection was performed in a minimal volume of medium with gentle swirling of culture flasks for about 15 min to enhance the contact between bacteria and host cells, resulting in efficient adhesion and internalization of rickettsiae. At this point, additional culture medium was added to each flask to bring the total volume to about 3 ml and cells were incubated at either 37 °C or 34 °C depending on the objective of the experiment for an additional 3 or 24 h as early and late time points of infection, respectively. For quantitative PCR, HMECs incubated with *R. conorii* for the first 15 min were used as a baseline control as described [24].

Infection of AAE2 cells was performed in 25cm^2^ flasks as described [24]. The L15B complete medium was replaced with the minimal volume of L15B infection medium prior to addition of *R. conorii*. The cells were infected with the inoculum of a pre-determined stock as described above, followed by gentle swirling and incubated for 15 min at 34 °C. Finally, 3 ml of fresh L15B medium was added to each flask and the cells were further incubated for 3 h and 24 h.

Total RNA isolation was carried out using Tri-Reagent (Molecular Research Center) following an optimized version of the manufacturer’s protocol. At each time point, culture medium was aspirated off carefully and the cells (HMECs or AAE2) infected with *R. conorii* were lysed in Tri-Reagent and processed for RNA isolation using our standard laboratory protocol. Total RNA thus obtained was subjected to DNase I treatment to eliminate genomic DNA contamination, purified by precipitation with 3 M sodium acetate pH 5.5 (Ambion) and glycogen (5 μg/mL) (Ambion), and dissolved in nuclease-free water. The quality of total RNA preparations was assessed on a Bioanalyzer (Agilent Technologies) and only samples with an RNA integrity number (RIN) score of ≥9.0 were used for sequencing.

### Library preparation and data analysis

RNA samples were enriched for bacterial coding and non-coding transcripts using Dynabeads Oligo (dT)_25_ (Thermo Fisher Scientific) and Ribo-Zero Gold rRNA Removal Kit (Epidemiology) (Illumina) to remove mRNAs (eukaryotic) and rRNAs (bacterial, eukaryotic, and mitochondrial), respectively. Enriched RNA from each biological replicate was then split into two equal aliquots, of which one was treated with Terminator 5′-Phosphate-dependent Exonuclease (TEX) to remove processed transcripts and designated as ‘+TEX’. The other untreated aliquot containing both primary (5′-triphosphate) and processed (5′-monophosphate) transcripts was designated as ‘-TEX’. Complementary DNA libraries for each sample were prepared independently using TruSeq RNA Sample Prep Kit following manufacturer instructions (Illumina). Strand-specific, paired end reads of 100 bases in length were sequenced on an Illumina HiSeq 1500 at the institutional Next Generation Sequencing core facility of the UTMB. A minimum of 70 million reads were sequenced from each biological replicate. The sequences were analyzed using the CLC Genomics Workbench 12.0.3 Microbial Genomics Module. Reads containing nucleotides below the quality threshold of 0.05 (using the modified Richard Mott algorithm) and those with two or more unknown nucleotides or sequencing adapters were trimmed out. The reads from each library were mapped to *R. conorii* genome (NC_003103.1) in PATRIC database in light of its consistency for the annotation of rickettsial genomes. The criteria for read mapping included an allowance of up to two mismatches per read and removal of all unmapped reads from the analysis [24]. Reads per kilobase per million of mapped reads (RKPM) were calculated using the formula: Total reads mapping to the gene (ORF) / [mapped reads (million) * gene length (kb)]. To avoid an intrinsic statistical bias, we calculated transcripts per million (TPM) for each expressed transcript (ORF) as described previously [25] using the formula: RKPM * 10^6^ / Ʃ RKPM.

### Quantitative RT-PCR

To confirm the expression profile of novel *Rc*_sRs and differentially expressed genes, we performed quantitative RT-PCR of three sRNAs (*Rc*_sR61, sR71, and sR74) and two rickettsial transcripts (*RC0149* and *RC0511*) during in vitro infection of human ECs and tick cells. One microgram of total RNA was reverse transcribed using random primers and high capacity reverse transcription kit (Invitrogen) according to the manufacturer’s protocol. SYBR green based RT-qPCR was performed using sRNA- or gene-specific primer pairs and rickettsial 16S RNA as an endogenous control to account for and nullify the differences in bacterial load between samples. The expression profile of *R. conorii* 6S RNA was assessed by a TaqMan assay described previously [22]. Total RNA was reverse transcribed as described above and custom synthesized 6S RNA primers and probe as well as the corresponding 16S RNA primers and probe were used to quantify transcript abundance at different times during the course of infection. Owing to the obligate intracellular lifestyle of rickettsiae, cells infected for only 15 min served as the baseline control and relative quantity was calculated as described below. The ^Δ^Ct values for *R. conorii-*infected cells at 3 h and 24 h were compared to those infected for 15 min (designated as the baseline control), which was assigned a value of 1. Relative expression was determined by comparative Ct (^−∆∆^Ct method) [94]. Briefly, expression of *R. conorii* genes or sRNAs was quantified using specific primers and rickettsial 16S rRNA as the housekeeping control. To obtain ^Δ^Ct values, the Ct values for target gene and sRNA at each time point were normalized to the Ct value for 16S RNA using the StepOne™ Plus software version 2.3. We determined relative expression of target sRNA or genes at 3 h and 24 h by comparing normalized target quantity at each time point to the normalized quantity in cells infected for 15 min (baseline control). The data thus obtained were plotted as the fold-change over basal expression [94]. The values from a minimum of three independent biological replicates processed as two technical replicates for each time point were analyzed by ^-^^∆∆^Ct method. All primers and probes used in this study are listed in Additional file 8.

### Identification of transcription start sites (TSSs)

Quality trimmed reads from +TEX and -TEX libraries mapping to *R. conorii* genome were used for the identification of TSSs using the program TSSAR (http://rna.tbi.univie.ac.at/TSSAR/) with default parameters [95]. The TSSs were classified as primary (within 250 nucleotides upstream of the gene translational start site), internal (within the coding gene), antisense (on the opposite strand of an annotated gene), or orphan (anywhere else including the intergenic region of the genome), depending on their genomic location with respect to the gene annotation. The antisense TSSs were further subdivided as ‘AiTSS’ (TSS on the opposite strand and within the coding ORF) and ‘AdTSS’ (TSS on the anti-sense strand and within 30 bp downstream of a stop codon of an annotated gene) depending on their genomic location.

### Statistical analysis

All quantitative RT-PCR experiments were performed on a minimum of three independent biological replicates with two technical replicates for each time point. The data was analyzed using GraphPad Prism and calculated as the mean ± standard error of the mean (SEM). Statistical analysis was performed using Mann-Whitney t-test with a significance threshold of *p* ≤ 0.05.

## Supplementary information

**Additional file 1 **Growth kinetics of *R. conorii* in HMECs infected and maintained at either 34 °C or 37 °C, and AAE2 cells infected and maintained at 34 °C, for 24 h post-infection.

**Additional file 2 **List of *R. conorii* coding genes and their expression during infection of HMECs and AAE2 cells, in vitro.

**Additional file 3 **Expression of genes involved in LPS biosynthesis and transport (A), type IV secretion system and known rickettsial effectors (B), and genes encoding ankyrin repeat proteins (C) in *R. conorii* during infection of HMECs and AAE2 cells, in vitro.

**Additional file 4 **List of different categories (Ad, Ai, I, O and IP) of transcription start sites identified by TSSAR in *R. conorii* genome during the infection of HMECs, in vitro.

**Additional file 5 **List of different categories (Ad, Ai, I, O and IP) of transcription start sites identified by TSSAR in *R. conorii* genome during the infection of AAE2 cells, in vitro.

**Additional file 6 **List of primary transcription start sites (pTSS) identified for *R. conorii* transcripts expressed during the infection of tick vector (AAE2 + *Rc*) and human host (HMEC+*Rc*) cells, in vitro.

**Additional file 7 **List of small RNAs identified in *R. conorii* genome.

**Additional file 8.** List of primers used in this study.

## Data Availability

The data from this study are included in this published article and its supplementary additional files. The raw sequencing data is deposited to the NCBI database under accession number PRJNA657619.

## Abbreviations

EC’s: Endothelial cells
ECFs: Extracytoplasmic functional sigma factors
HMECs: Human microvascular endothelial cells
MOI: Multiplicity of infection
mRNA: Messenger ribonucleic acid
TSS: Transcription start site
*Rc*_sR’s: *Rickettsia conorii* small RNA
RT-PCR: Reverse transcriptase polymerase chain reaction
RKPM: Reads per kilobase million
sRNA: Small ribonucleic acid
TEX: Terminator 5′-Phosphate-dependent Exonuclease
TPM: Transcripts per million

## References

[1] Azad AF (2007). Pathogenic rickettsiae as bioterrorism agents. Clin Infect Dis.

[2] Sahni A, Fang R, Sahni SK, Walker DH. Pathogenesis of rickettsial diseases: pathogenic and immune mechanisms of an endotheliotropic infection. Annu Rev Pathol. 2018. 10.1146/annurev-pathmechdis-012418-012800 PMC6505701 30148688

[3] Parola P, Paddock CD, Socolovschi C, Labruna MB, Mediannikov O, Kernif T (2013). Update on tick-borne rickettsioses around the world: a geographic approach. Clin Microbiol Rev.

[4] Sahni SK, Narra HP, Sahni A, Walker DH (2013). Recent molecular insights into rickettsial pathogenesis and immunity. Future Microbiol.

[5] Ellison DW, Clark TR, Sturdevant DE, Virtaneva K, Hackstadt T (2009). Limited transcriptional responses of *Rickettsia rickettsii* exposed to environmental stimuli. PLoS One.

[6] Dreher-Lesnick SM, Ceraul SM, Rahman MS, Azad AF (2008). Genome-wide screen for temperature-regulated genes of the obligate intracellular bacterium, *Rickettsia typhi*. BMC Microbiol.

[7] Galletti MF, Fujita A, Nishiyama MY, Malossi CD, Pinter A, Soares JF (2013). Natural blood feeding and temperature shift modulate the global transcriptional profile of *Rickettsia rickettsii* infecting its tick vector. PLoS One.

[8] Galletti MF, Fujita A, Rosa RD, Martins LA, Soares HS, Labruna MB (2016). Virulence genes of *Rickettsia rickettsii* are differentially modulated by either temperature upshift or blood-feeding in tick midgut and salivary glands. Parasit Vectors.

[9] Nelson CM, Herron MJ, Wang XR, Baldridge GD, Oliver JD, Munderloh UG (2020). Global transcription profiles of *Anaplasma phagocytophilum* at key stages of infection in tick and human cell lines and granulocytes. Front Vet Sci.

[10] Kuriakose JA, Miyashiro S, Luo T, Zhu B, McBride JW (2011). *Ehrlichia chaffeensis* transcriptome in mammalian and arthropod hosts reveals differential gene expression and post transcriptional regulation. PLoS One.

[11] de Silva AM, Fikrig E (1997). Arthropod- and host-specific gene expression by *Borrelia burgdorferi*. J Clin Invest.

[12] Nelson CM, Herron MJ, Felsheim RF, Schloeder BR, Grindle SM, Chavez AO (2008). Whole genome transcription profiling of *Anaplasma phagocytophilum* in human and tick host cells by tiling array analysis. BMC Genomics.

[13] Tilly K, Bestor A, Rosa PA (2016). Functional equivalence of OspA and OspB, but not OspC, in tick colonization by *Borrelia burgdorferi*. Infect Immun.

[14] Gilmore RD, Piesman J (2000). Inhibition of *Borrelia burgdorferi* migration from the midgut to the salivary glands following feeding by ticks on OspC-immunized mice. Infect Immun.

[15] Neelakanta G, Li X, Pal U, Liu X, Beck DS, DePonte K (2007). Outer surface protein B is critical for *Borrelia burgdorferi* adherence and survival within *Ixodes* ticks. PLoS Pathog.

[16] Hellwage J, Meri T, Heikkila T, Alitalo A, Panelius J, Lahdenne P (2001). The complement regulator factor H binds to the surface protein OspE of *Borrelia burgdorferi*. J Biol Chem.

[17] Pal U, Fikrig E (2003). Adaptation of *Borrelia burgdorferi* in the vector and vertebrate host. Microbes Infect.

[18] Popitsch N, Bilusic I, Rescheneder P, Schroeder R, Lybecker M (2017). Temperature-dependent sRNA transcriptome of the Lyme disease spirochete. BMC Genomics.

[19] Cheah HL, Raabe CA, Lee LP, Rozhdestvensky TS, Citartan M, Ahmed SA (2018). Bacterial regulatory RNAs: complexity, function, and putative drug targeting. Crit Rev Biochem Mol Biol.

[20] Bojanovic K, D’Arrigo I, Long KS. Global transcriptional responses to osmotic, oxidative, and imipenem stress conditions in *Pseudomonas putida*. Appl Environ Microbiol. 2017;83. 10.1128/AEM.03236-16 PMC5359501 28130298

[21] Westermann AJ, Forstner KU, Amman F, Barquist L, Chao Y, Schulte LN (2016). Dual RNA-seq unveils noncoding RNA functions in host-pathogen interactions. Nature..

[22] Schroeder CL, Narra HP, Rojas M, Sahni A, Patel J, Khanipov K (2015). Bacterial small RNAs in the genus *Rickettsia*. BMC Genomics.

[23] Schroeder CL, Narra HP, Sahni A, Rojas M, Khanipov K, Patel J (2016). Identification and characterization of novel small RNAs in *Rickettsia prowazekii*. Front Microbiol.

[24] Narra HP, Schroeder CL, Sahni A, Rojas M, Khanipov K, Fofanov Y (2016). Small regulatory RNAs of *Rickettsia conorii*. Sci Rep.

[25] Schroeder CLC, Narra HP, Sahni A, Khanipov K, Patel J, Fofanov Y (2017). Transcriptional profiling of *Rickettsia prowazekii* coding and non-coding transcripts during in vitro host-pathogen and vector-pathogen interactions. Ticks Tick Borne Dis.

[26] Kumar N, Lin M, Zhao X, Ott S, Santana-Cruz I, Daugherty S (2016). Efficient enrichment of bacterial mRNA from host-bacteria total RNA samples. Sci Rep.

[27] Niebylski ML, Peacock MG, Schwan TG (1999). Lethal effect of *Rickettsia rickettsii* on its tick vector (*Dermacentor andersoni*). Appl Environ Microbiol.

[28] Patel JG, Narra HP, Sepuru KM, Sahni A, Golla SR, Sahni A (2020). Evolution, purification, and characterization of RC0497: a peptidoglycan amidase from the prototypical spotted fever species *Rickettsia conorii*. Biol Chem.

[29] Zhao Y, Fang R, Zhang J, Zhang Y, Bechelli J, Smalley C (2020). Quantitative proteomics of the endothelial secretome identifies RC0497 as diagnostic of acute rickettsial spotted fever infections. Am J Pathol.

[30] Audia JP, Patton MC, Winkler HH (2008). DNA microarray analysis of the heat shock transcriptome of the obligate intracytoplasmic pathogen *Rickettsia prowazekii*. Appl Environ Microbiol.

[31] Ogawa M, Renesto P, Azza S, Moinier D, Fourquet P, Gorvel JP (2007). Proteome analysis of *Rickettsia felis* highlights the expression profile of intracellular bacteria. Proteomics..

[32] Moran NA (1996). Accelerated evolution and Muller's rachet in endosymbiotic bacteria. Proc Natl Acad Sci U S A.

[33] Fares MA, Moya A, Barrio E (2005). Adaptive evolution in GroEL from distantly related endosymbiotic bacteria of insects. J Evol Biol.

[34] Sabater-Munoz B, Prats-Escriche M, Montagud-Martinez R, Lopez-Cerdan A, Toft C, Aguilar-Rodriguez J (2015). Fitness trade-offs determine the role of the molecular chaperonin GroEL in buffering mutations. Mol Biol Evol.

[35] Yamanaka K, Fang L, Inouye M (1998). The CspA family in *Escherichia coli:* multiple gene duplication for stress adaptation. Mol Microbiol.

[36] Schmid B, Klumpp J, Raimann E, Loessner MJ, Stephan R, Tasara T (2009). Role of cold shock proteins in growth of listeria monocytogenes under cold and osmotic stress conditions. Appl Environ Microbiol.

[37] Caballero CJ, Menendez-Gil P, Catalan-Moreno A, Vergara-Irigaray M, Garcia B, Segura V (2018). The regulon of the RNA chaperone CspA and its auto-regulation in *Staphylococcus aureus*. Nucleic Acids Res.

[38] Loepfe C, Raimann E, Stephan R, Tasara T (2010). Reduced host cell invasiveness and oxidative stress tolerance in double and triple csp gene family deletion mutants of *Listeria monocytogenes*. Foodborne Pathog Dis.

[39] Wang Z, Liu W, Wu T, Bie P, Wu Q (2016). RNA-seq reveals the critical role of CspA in regulating *Brucella melitensis* metabolism and virulence. Sci China Life Sci.

[40] Wang Z, Wang S, Wu Q (2014). Cold shock protein a plays an important role in the stress adaptation and virulence of *Brucella melitensis*. FEMS Microbiol Lett.

[41] Lamason RL, Welch MD (2017). Actin-based motility and cell-to-cell spread of bacterial pathogens. Curr Opin Microbiol.

[42] Harris EK, Jirakanwisal K, Verhoeve VI, Fongsaran C, Suwanbongkot C, Welch MD, et al. Role of Sca2 and RickA in the dissemination of *Rickettsia parkeri* in *Amblyomma maculatum*. Infect Immun. 2018;86. 10.1128/IAI.00123-18 PMC5964526 29581194

[43] Audoly G, Vincentelli R, Edouard S, Georgiades K, Mediannikov O, Gimenez G (2011). Effect of rickettsial toxin VapC on its eukaryotic host. PLoS One.

[44] Botelho-Nevers E, Edouard S, Leroy Q, Raoult D (2012). Deleterious effect of ciprofloxacin on *Rickettsia conorii*-infected cells is linked to toxin-antitoxin module up-regulation. J Antimicrob Chemother.

[45] Keren I, Shah D, Spoering A, Kaldalu N, Lewis K (2004). Specialized persister cells and the mechanism of multidrug tolerance in *Escherichia coli*. J Bacteriol.

[46] Wang X, Wood TK (2011). Toxin-antitoxin systems influence biofilm and persister cell formation and the general stress response. Appl Environ Microbiol.

[47] Kaur SJ, Rahman MS, Ammerman NC, Beier-Sexton M, Ceraul SM, Gillespie JJ (2012). TolC-dependent secretion of an ankyrin repeat-containing protein of *Rickettsia typhi*. J Bacteriol.

[48] Lehman SS, Noriea NF, Aistleitner K, Clark TR, Dooley CA, Nair V, et al. The rickettsial ankyrin repeat protein 2 is a type IV secreted effector that associates with the endoplasmic reticulum. MBio. 2018;9. 10.1128/mBio.00975-18 PMC6020290 29946049

[49] Aistleitner K, Clark T, Dooley C, Hackstadt T (2020). Selective fragmentation of the trans-Golgi apparatus by *Rickettsia rickettsii*. PLoS Pathog.

[50] VieBrock L, Evans SM, Beyer AR, Larson CL, Beare PA, Ge H (2014). *Orientia tsutsugamushi* ankyrin repeat-containing protein family members are type 1 secretion system substrates that traffic to the host cell endoplasmic reticulum. Front Cell Infect Microbiol.

[51] Evans SM, Rodino KG, Adcox HE, Carlyon JA (2018). *Orientia tsutsugamushi* uses two Ank effectors to modulate NF-kappaB p65 nuclear transport and inhibit NF-kappaB transcriptional activation. PLoS Pathog.

[52] Beyer AR, VieBrock L, Rodino KG, Miller DP, Tegels BK, Marconi RT (2015). *Orientia tsutsugamushi* strain Ikeda ankyrin repeat-containing proteins recruit SCF1 ubiquitin ligase machinery via poxvirus-like F-box motifs. J Bacteriol.

[53] Hase CC, Finkelstein RA (1993). Bacterial extracellular zinc-containing metalloproteases. Microbiol Rev.

[54] Bozhokina E, Kever L, Khaitlina S. The *Serratia grimesii* outer membrane vesicles-associated grimelysin triggers bacterial invasion of eukaryotic cells. Cell Biol Int. 2020. 10.1002/cbin.11435 32749752

[55] Valeri M, Rossi Paccani S, Kasendra M, Nesta B, Serino L, Pizza M (2015). Pathogenic *E coli* exploits SslE mucinase activity to translocate through the mucosal barrier and get access to host cells. PLoS One.

[56] Cerveny L, Straskova A, Dankova V, Hartlova A, Ceckova M, Staud F (2013). Tetratricopeptide repeat motifs in the world of bacterial pathogens: role in virulence mechanisms. Infect Immun.

[57] Walburger A, Koul A, Ferrari G, Nguyen L, Prescianotto-Baschong C, Huygen K (2004). Protein kinase G from pathogenic mycobacteria promotes survival within macrophages. Science..

[58] Allmond LR, Karaca TJ, Nguyen VN, Nguyen T, Wiener-Kronish JP, Sawa T (2003). Protein binding between PcrG-PcrV and PcrH-PopB/PopD encoded by the pcrGVH-popBD operon of the *Pseudomonas aeruginosa* type III secretion system. Infect Immun.

[59] Mohapatra NP, Soni S, Rajaram MV, Dang PM, Reilly TJ, El-Benna J (2010). *Francisella* acid phosphatases inactivate the NADPH oxidase in human phagocytes. J Immunol.

[60] Wurtzel O, Sapra R, Chen F, Zhu Y, Simmons BA, Sorek R (2010). A single-base resolution map of an archaeal transcriptome. Genome Res.

[61] Wade JT, Grainger DC (2014). Pervasive transcription: illuminating the dark matter of bacterial transcriptomes. Nat Rev Microbiol.

[62] He Y, Vogelstein B, Velculescu VE, Papadopoulos N, Kinzler KW (2008). The antisense transcriptomes of human cells. Science..

[63] Thomason MK, Bischler T, Eisenbart SK, Forstner KU, Zhang A, Herbig A (2015). Global transcriptional start site mapping using differential RNA sequencing reveals novel antisense RNAs in *Escherichia coli*. J Bacteriol.

[64] Zhukova A, Fernandes LG, Hugon P, Pappas CJ, Sismeiro O, Coppee JY (2017). Genome-wide transcriptional start site mapping and sRNA identification in the pathogen *Leptospira interrogans*. Front Cell Infect Microbiol.

[65] Adams PP, Flores Avile C, Popitsch N, Bilusic I, Schroeder R, Lybecker M (2017). In vivo expression technology and 5′ end mapping of the *Borrelia burgdorferi* transcriptome identify novel RNAs expressed during mammalian infection. Nucleic Acids Res.

[66] Sharma CM, Hoffmann S, Darfeuille F, Reignier J, Findeiss S, Sittka A (2010). The primary transcriptome of the major human pathogen *Helicobacter pylori*. Nature..

[67] Raghavan R, Sloan DB, Ochman H. Antisense transcription is pervasive but rarely conserved in enteric bacteria. MBio. 2012;3. 10.1128/mBio.00156-12 PMC3419515 22872780

[68] Chatterjee A, Johnson CM, Shu CC, Kaznessis YN, Ramkrishna D, Dunny GM (2011). Convergent transcription confers a bistable switch in *Enterococcus faecalis* conjugation. Proc Natl Acad Sci U S A.

[69] Lee EJ, Groisman EA (2010). An antisense RNA that governs the expression kinetics of a multifunctional virulence gene. Mol Microbiol.

[70] Chakravarty S, Masse E (2019). RNA-dependent regulation of virulence in pathogenic bacteria. Front Cell Infect Microbiol.

[71] Saberi F, Kamali M, Najafi A, Yazdanparast A, Moghaddam MM (2016). Natural antisense RNAs as mRNA regulatory elements in bacteria: a review on function and applications. Cell Mol Biol Lett.

[72] Wagner EG (2009). Kill the messenger: bacterial antisense RNA promotes mRNA decay. Nat Struct Mol Biol.

[73] Yang Z, Jin X, Rao X, Hu F (2011). A natural antisense transcript regulates mucD gene expression and biofilm biosynthesis in *Pseudomonas aeruginosa*. Mikrobiologiia..

[74] Brophy JA, Voigt CA (2016). Antisense transcription as a tool to tune gene expression. Mol Syst Biol.

[75] Cai J, Pang H, Wood DO, Winkler HH (1995). The citrate synthase-encoding gene of *Rickettsia prowazekii* is controlled by two promoters. Gene..

[76] Thairu MW, Hansen AK. Changes in aphid host plant diet influence the small-RNA expression profiles of its obligate nutritional symbiont, *Buchnera*. mBio. 2019;10. 10.1128/mBio.01733-19 PMC6867890 31744912

[77] Thairu MW, Cheng S, Hansen AK (2018). A sRNA in a reduced mutualistic symbiont genome regulates its own gene expression. Mol Ecol.

[78] Rau MH, Bojanovic K, Nielsen AT, Long KS (2015). Differential expression of small RNAs under chemical stress and fed-batch fermentation in *E coli*. BMC Genomics.

[79] Wassarman KM. 6S RNA, a global regulator of transcription. Microbiol Spectr. 2018;6. 10.1128/microbiolspec.rwr-0019-2018 PMC6013841 29916345

[80] Warrier I, Hicks LD, Battisti JM, Raghavan R, Minnick MF (2014). Identification of novel small RNAs and characterization of the 6S RNA of *Coxiella burnetii*. PLoS One.

[81] Darby AC, Armstrong SD, Bah GS, Kaur G, Hughes MA, Kay SM (2012). Analysis of gene expression from the *Wolbachia* genome of a filarial nematode supports both metabolic and defensive roles within the symbiosis. Genome Res.

[82] Drecktrah D, Hall LS, Brinkworth AJ, Comstock JR, Wassarman KM, Samuels DS (2020). Characterization of 6S RNA in the Lyme disease spirochete. Mol Microbiol.

[83] Trotochaud AE, Wassarman KM (2004). 6S RNA function enhances long-term cell survival. J Bacteriol.

[84] Sharma UK, Chatterji D (2010). Transcriptional switching in Escherichia coli during stress and starvation by modulation of sigma activity. FEMS Microbiol Rev.

[85] Chevalier S, Bouffartigues E, Bazire A, Tahrioui A, Duchesne R, Tortuel D (1862). Extracytoplasmic function sigma factors in *Pseudomonas aeruginosa*. Biochim Biophys Acta Gene Regul Mech.

[86] Helmann JD (2002). The extracytoplasmic function (ECF) sigma factors. Adv Microb Physiol.

[87] Martinez-Salazar JM, Salazar E, Encarnacion S, Ramirez-Romero MA, Rivera J (2009). Role of the extracytoplasmic function sigma factor RpoE4 in oxidative and osmotic stress responses in *Rhizobium etli*. J Bacteriol.

[88] Munderloh UG, Kurtti TJ (1989). Formulation of medium for tick cell culture. Exp Appl Acarol.

[89] Narra HP, Sahni A, Khanipov K, Fofanov Y, Sahni SK. Global transcriptomic profiling of pulmonary gene expression in an experimental murine model of *Rickettsia conorii* infection. Genes (Basel). 2019:10. 10.3390/genes10030204 PMC6470625 30857242

[90] Ammerman NC, Beier-Sexton M, Azad AF. Laboratory maintenance of *Rickettsia rickettsii*. Curr Protoc Microbiol. 2008;Chapter 3:Unit 3A 5. 10.1002/9780471729259.mc03a05s11 PMC2725428 19016440

[91] Alhassan A, Liu H, McGill J, Cerezo A, Jakkula L, Nair ADS, et al. *Rickettsia rickettsii* whole-cell antigens offer protection against rocky mountain spotted fever in the canine host. Infect Immun. 2019;87. 10.1128/IAI.00628-18 PMC6346123 30396898

[92] Labruna MB, Whitworth T, Horta MC, Bouyer DH, McBride JW, Pinter A (2004). *Rickettsia* species infecting *Amblyomma cooperi* ticks from an area in the state of Sao Paulo, Brazil, where Brazilian spotted fever is endemic. J Clin Microbiol.

[93] Sahni A, Patel J, Narra HP, Schroeder CLC, Walker DH, Sahni SK (2017). Fibroblast growth factor receptor-1 mediates internalization of pathogenic spotted fever rickettsiae into host endothelium. PLoS One.

[94] Schmittgen TD, Livak KJ (2008). Analyzing real-time PCR data by the comparative C(T) method. Nat Protoc.

[95] Amman F, Wolfinger MT, Lorenz R, Hofacker IL, Stadler PF, Findeiss S (2014). TSSAR: TSS annotation regime for dRNA-seq data. BMC Bioinformatics.

