# Supplementary material for: Comparative transcriptomic analysis of Rickettsia conorii during in vitro infection of human and tick host cells
Source: BMC Genomics. 2020 Sep 25;21:665. doi: 10.1186/s12864-020-07077-w (PMC7519539; doi:10.1186/s12864-020-07077-w)
Supplement: Supplementary file 1 — Additional file 1 Growth kinetics of R. conorii in HMECs infected and maintained at either 34 °C or 37 °C, and AAE2 cells infected and maintained at 34 °C, for 24 h post-infection. [file 12864_2020_7077_MOESM1_ESM.pptx]

## Slide 1
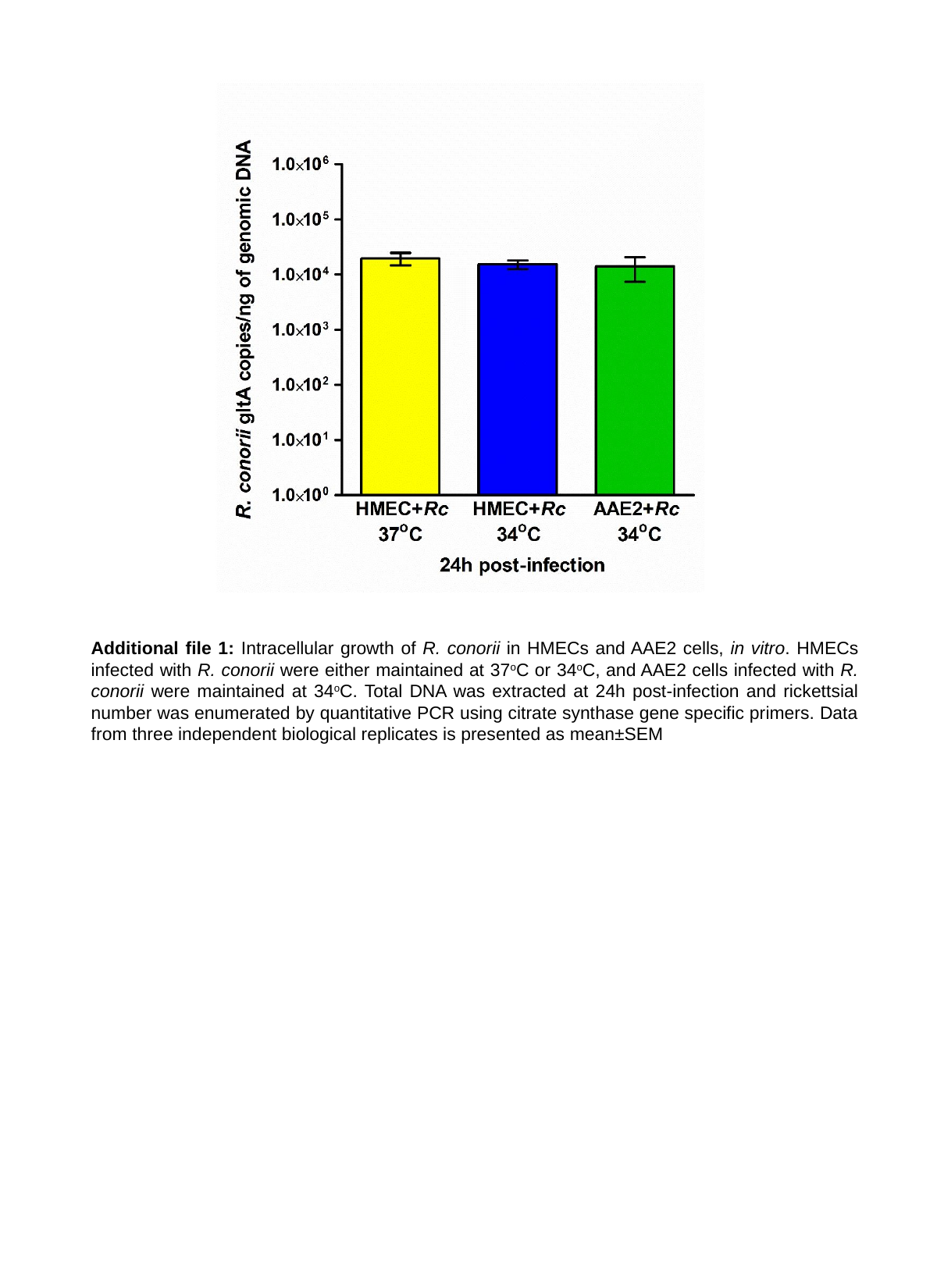

Additional file 1: Intracellular growth of R. conorii in HMECs and AAE2 cells, in vitro. HMECs infected with R. conorii were either maintained at 37oC or 34oC, and AAE2 cells infected with R. conorii were maintained at 34oC. Total DNA was extracted at 24h post-infection and rickettsial number was enumerated by quantitative PCR using citrate synthase gene specific primers. Data from three independent biological replicates is presented as mean±SEM
